# Enhanced visualization of influenza A virus entry into living cells using virus-view atomic force microscopy

**DOI:** 10.1073/pnas.2500660122

**Published:** 2025-09-18

**Authors:** Aiko Yoshida, Yoshitsugu Uekusa, Takeshi Suzuki, Michael Bauer, Nobuaki Sakai, Yohei Yamauchi

**Affiliations:** ^a^Department of Cell Physiology, Faculty of Medicine and Graduate School of Medicine, Hokkaido University, Sapporo 060-8638, Japan; ^b^R&D Group, Olympus Corporation, Hachioji 192-8512, Japan; ^c^Department of Virology, Graduate School of Medicine, Nagoya University, Nagoya 466-8550, Japan; ^d^Molecular Medicine Laboratory, Institute of Pharmaceutical Sciences, Department of Chemistry and Applied Biosciences, Eidgenössische Technische Hochschule Zürich, Zürich 8093, Switzerland; ^e^Department of Molecular Life Sciences, University of Zurich, Zurich 8057, Switzerland; ^f^School of Cellular and Molecular Medicine, Faculty of Health and Life Sciences, University of Bristol, Bristol BS8 1TD, United Kingdom

**Keywords:** live-cell atomic force microscopy, influenza A virus, membrane dynamics, virus–cell interactions, virus cell entry

## Abstract

Influenza A virus (IAV) entry into host cells begins with interactions between the viral envelope proteins hemagglutinin (HA)/neuraminidase (NA) and sialic acid moieties on the cell plasma membrane. These interactions drive IAV’s lateral diffusion along the cell membrane and trigger membrane morphological changes required for endocytosis. However, directly visualizing these dynamic processes, which are crucial for IAV entry, has been challenging using conventional microscopy techniques. In this study, we enabled live-cell observation of nanoscale morphological dynamics of IAV and the cell membrane by reducing the mechanical invasiveness of atomic force microscopy (AFM). A customised cantilever with less than half the spring constant of conventional cantilevers enabled virus-view AFM imaging that preserved IAV–membrane interactions. By combining virus-view AFM with confocal microscopy, we performed correlative morphological and fluorescence observations of IAV lateral diffusion and endocytosis in living cells. Variations in diffusion coefficients of single virions suggested heterogeneity in sialic acid density on the cell membrane. NA inhibition decreased diffusion coefficients, while reduced sialic acid density increased them. The timing of clathrin accumulation at virion binding sites coincided with a decrease in diffusion coefficients, a relationship that was maintained independent of NA activity or sialic acid density. As clathrin assembly progressed, ~100-nm-high membrane bulges emerged adjacent to the virus, culminating in the complete membrane envelopment of the virus at peak clathrin accumulation. Our virus-view AFM will deepen our understanding of various virus–cell interactions, facilitate the evaluation of drug effects and promote future translational research.

## Significance

Influenza A viruses (IAVs) continue to cause epidemics worldwide due to their high mutability. Nevertheless, the initial step of infection, viral uptake into cells, has been challenging to observe directly with conventional microscopy techniques. Here, we developed a hybrid imaging system combining atomic force microscopy and confocal microscopy with enhanced mechanical functionality and minimal invasiveness to directly visualize nanoscale dynamics of IAV and cell membranes during viral uptake into living cells. This system enables the analysis of IAV lateral diffusion resulting from IAV–membrane interactions and characteristic membrane morphological changes induced by IAV during endocytosis. Our approach offers a method to rapidly assess the impact of viral mutations on host cell entry, which is critical for understanding emerging IAV variants.

Influenza A virus (IAV) is an enveloped RNA virus with two key surface glycoproteins: hemagglutinin (HA) and neuraminidase (NA). The virus surface contains 300 to 400 HA and 40 to 50 NA molecules (1). IAV envelope proteins comprise at least 18 HA and 11 NA subtypes (2), which enable IAV to infect various host species including humans, birds, pigs, bats, and other animals (3). These envelope proteins play crucial roles in IAV infection of host cells.

They interact with sialic acids on cell surface glycolipids and glycoproteins (4) or with major histocompatibility complex class II (MHC class II) molecules (5–7). HA binds to sialic acids at the terminal ends of glycan chains on the cell surface. The HA–sialic acid interactions are inherently weak, with dissociation constants typically in the millimolar range (0.9 to 68.4 × 10^−3^ M) (8–10). However, multivalent binding of multiple HAs to sialic acids enables IAV to stably adhere to the cell membrane (11, 12). Meanwhile, NA catalyzes the cleavage of sialic acids (13), inhibiting stable adhesion of IAV to the cell membrane. Through these mechanisms, HA and NA effectively regulate the attachment and detachment of IAV to the cell membrane.

The competitive action between HA and NA allows IAV to diffuse laterally along the cell membrane surface topology (14). This lateral diffusion represents a critical dynamic macroscopic phenomenon reflecting virus–membrane interactions. However, conventional microscopy techniques have struggled to detect IAV movement on the 10-nm-thick cell membrane, resulting in limited visualization success (15–18).

HA-NA-sialic acid interactions also trigger endocytosis involving morphological changes of the cell membrane. When diffusing IAV binds to functional receptors such as EGFR (19) and Cav1.2 (20) through sialic acids, it initiates the recruitment and assembly of the endocytic machinery including clathrin, actin, and dynamin. IAV utilizes multiple entry pathways including clathrin-mediated endocytosis (CME), macropinocytosis, and both clathrin-independent and dynamin-independent mechanisms (16, 21–23).

IAV primarily utilizes CME for cellular entry (16, 21). Previous imaging of membrane dynamics using atomic force microscopy (AFM) has revealed that in IAV-free CME, clathrin-coated membrane invaginations (pits) larger than 100 nm in diameter form (24, 25). This is accompanied by the emergence of actin-dependent membrane bulges that develop on one side of the pit and eventually lead to its closure. Although electron microscopy has provided morphological snapshots of pits during IAV internalization (26), the membrane dynamics during IAV internalization via CME have yet to be successfully visualized.

AFM enables mechanical imaging of sample morphology with nanometer-scale resolution (27, 28). Since the development of high-speed AFM in 2001 (29), this technique has contributed significantly to molecular dynamics analysis (30–36). Additionally, the advent of cell-imaging AFM in 2013 has enabled advances in membrane dynamics analysis (37, 38). The integration of cell-imaging AFM combined with confocal microscopy has provided unique capabilities for observing nanoscale membrane morphological changes in living cells (24, 25). Despite these advances, a major challenge persists: the mechanical interference of the cantilever with biological samples. Visualizing the dynamic processes of IAV lateral diffusion and internalization requires an innovative technology capable of simultaneously observing the nanoscale morphology of the 10-nm-thick cell membrane and the 100-nm spherical IAV interacting with cell surface sialic acid-bearing glycolipids and proteins. Given that multivalent IAV–membrane interaction forces are relatively weak, ranging from 10 to 25 pN (39), achieving low-invasive imaging capabilities is critical.

In this study, we address and overcome the challenge of mechanical interference by enhancing the low invasiveness of AFM through the use of a customised soft cantilever. In combination with confocal microscopy, low-invasive AFM enables simultaneous live-cell imaging of both morphology and fluorescence. The redesigned cantilever minimizes disruption of IAV–membrane interactions, allowing accurate observation of viral dynamics. Using this system, we investigated the lateral diffusion of single IAV particles under various conditions, including NA inhibition, reduced cell surface sialic acid density, and different viral subtypes. We also analyzed membrane morphological changes before and during IAV endocytosis. While fluorescently labeled IAV was primarily used, we also demonstrate our AFM’s capability to track unlabeled viruses. This virus-view dual confocal and AFM, called ViViD-AFM, enables correlative morphological and fluorescence imaging of IAV–membrane dynamics, providing nanoscopic insights into HA-NA-sialic acid interactions.

## Results

**Fig. 1.**
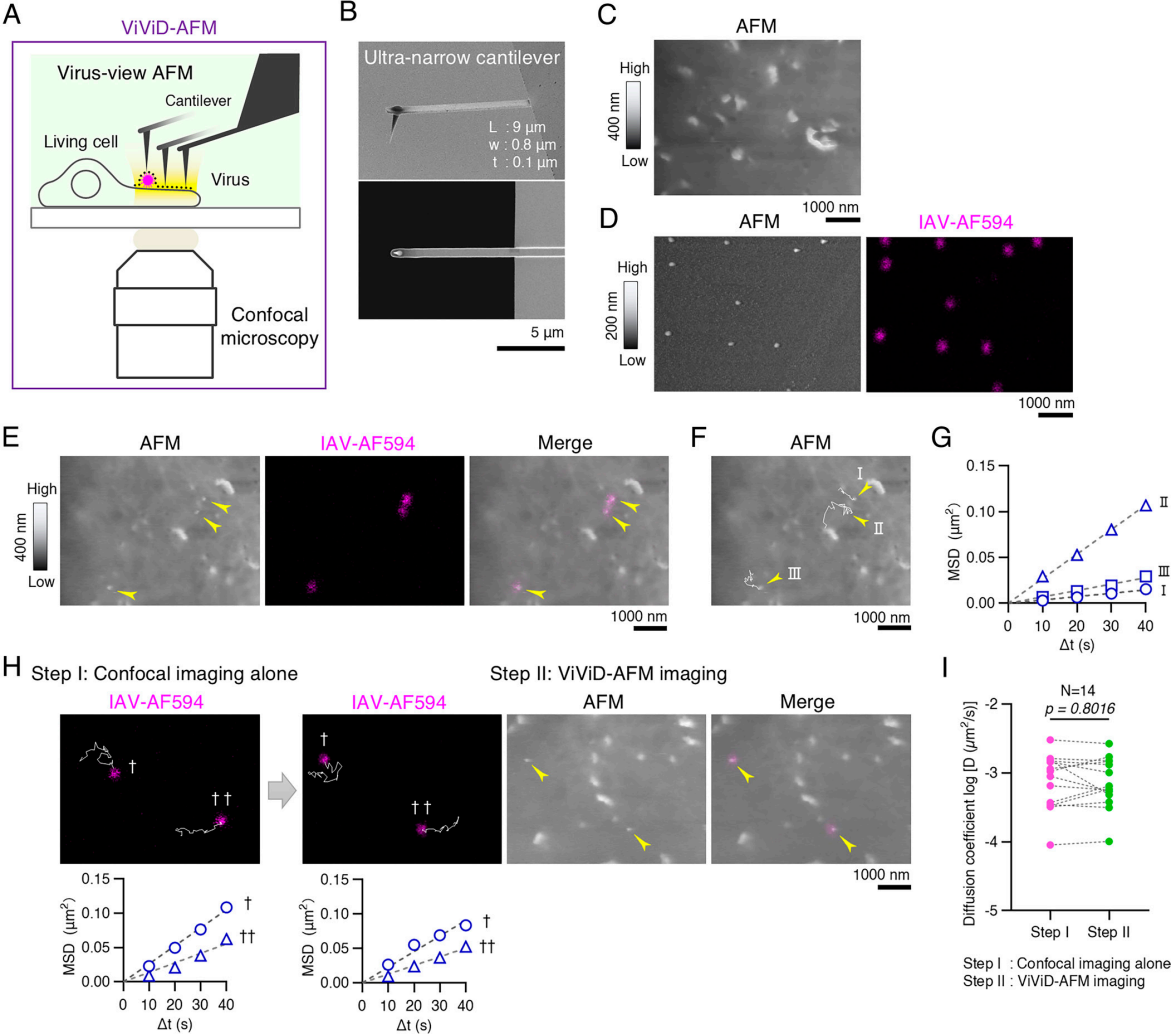
Establishment of virus-view AFM for IAV cell entry studies in MDCK cells. (*A*) Schematic of virus-view dual confocal and AFM (ViViD-AFM) showing simultaneous morphological and fluorescence imaging in liquid medium. The cantilever of virus-view AFM scans the surface of the living cell and the virion to provide morphological images while the confocal microscope detects the fluorescence signals. (*B*) Scanning electron microscopy images of the ultra-narrow cantilever. Side view (*Top*) and bottom view from the tip side (*Bottom*). (Scale bar, 5 µm.) (*C*) Virus-view AFM topographic image of the live MDCK cell surface at 27 °C. Brighter areas indicate higher regions; darker areas indicate lower regions. (*D*) Morphological (*Left*) and fluorescence (*Right*) images of glass-bound Alexa-Fluor 594-labeled IAV/WSN virions (IAV-AF594) acquired by ViViD-AFM. AFM height range: 200 nm. (*E*) ViViD-AFM imaging of cell surface IAV-AF594 virions (arrowheads) at 27 °C showing morphology (*Left*), fluorescence (*Middle*), and merged images (*Right*). (*F*) Tracking of cell surface IAV-AF594 virions at 10-s intervals. Diffusion trajectories of three IAV virions from panel (*E*) over 260 s, superimposed on the image. (*G*) Quantification of IAV-AF594 diffusion. Mean square displacement (MSD) plots for the three virions (I, II, and III) from panel (*F*) with dashed lines indicating two-dimensional free diffusion. Diffusion coefficients (*D*) calculated from MSD plots over 26 frames (260 s). (*H* and *I*) Assessment of ViViD-AFM imaging effects on IAV cell surface diffusion. Individual IAV-AF594 virions were tracked for 30 frames (300 s) each for Step I (confocal imaging alone) and Step II (ViViD-AFM imaging) at 27 °C at 10-s intervals. Diffusion coefficients of the identical virions were compared between steps I and II. (*H*) Representative diffusion analysis showing virion trajectories superimposed on fluorescence images (*Top*) and corresponding MSD plots (*Bottom*) for steps I (*Left*) and II (*Right*). (*I*) Diffusion coefficients of cell surface IAV-AF594 virions (N = 14) for steps I and II. Dashed lines connect measurements of identical virions. (*C*–*F* and *H*) (Scale bar, 1,000 nm. (*C*, *E*, *F*, and *H*) Height range: 400 nm.)

Fig. 1*A* shows the hybrid imaging system we used in this study that combines minimally invasive AFM (virus-view AFM) with confocal microscopy.

### Design of Virus-View AFM for Imaging of IAV–Cell Interactions.

In this study, we used Madin-Darby canine kidney (MDCK) cells due to their high susceptibility to a wide range of IAV strains (40). The interaction forces between IAV and MDCK cells are relatively weak; single-virus force spectroscopy has shown that the multivalent binding between HA and cellular sialic acid receptors measures only 10 to 25 pN at 27 °C (39). These weak interaction forces present a significant challenge for AFM imaging as conventional AFM systems exert forces during scanning (hereafter referred to as “peak force”) that can disrupt such delicate biological interactions, limiting the ability to perform truly noninvasive observations.

In our previous AFM study of the cell membrane in aqueous solution (24), we used an ultra-short cantilever with a length (L) of 9.0 µm, width (w) of 2.0 µm, and thickness (t) of 0.1 µm, yielding a spring constant of 0.1 N/m and a resonance frequency of over 300 kHz in liquid. The cantilever’s liquid resonance frequency of above 300 kHz is a necessary characteristic for video observation using AFM. However, based on the spring constant and the AFM control parameters, the minimum peak force exerted during scanning was estimated to be 25 pN. This level of force approaches the upper end of the multivalent interaction forces between IAV and the cell membrane (10 to 25 pN), which could potentially disrupt these interactions during imaging.

The spring constant ($k_{c}$) and resonance frequency ($f_{c}$) of a cantilever are related to its shape (length L, width w, thickness t) as follows:

[1] $$k_{c}\propto wt^{3}/L^{3},$$

[2] $$f_{c}\propto t/L^{2}.$$

To address this concern, we designed an “ultra-narrow” cantilever with a reduced spring constant, based on Eqs. **1** and 2. While reducing the spring constant was essential for minimizing invasiveness, we also needed to maintain a high resonance frequency of the cantilever in liquid to enable real-time imaging of cell membrane and virus movement. The redesigned cantilever features a length (L) of 9.0 μm, width (w) of 0.8 μm, and thickness (t) of 0.1 μm (Fig. 1*B*), achieving a spring constant of 0.04 N/m—approximately 60% lower than the previous designs—while preserving a resonance frequency above 300 kHz in liquid. This configuration reduced the minimum peak force to 10 pN, matching the lower threshold of virus–cell interaction forces. Hence, we termed this minimally invasive AFM system “virus-view AFM”.

### Establishment of ViViD-AFM for IAV Cell Entry Studies.

To evaluate whether virus-view AFM was suitable for detecting the cell surface morphology of live cultured cells, first, MDCK cells were imaged using AFM at 10-s intervals. The cell surface morphology within the field of view (6.0 × 4.5 µm) revealed numerous dynamic membrane protrusions of varying size and shape (Fig. 1*C* and Movie S1). These protrusions were smaller than those previously reported in A549 cells (41) and exhibited continuous morphological changes over time (*SI Appendix*, Fig. S1*A* and Movie S1). Virus-view AFM imaging of MDCK cells stably expressing the filamentous actin marker EGFP-Lifeact showed that the protrusions measured 100 to 200 nm in height and correlated with EGFP-Lifeact fluorescence intensity (r = 0.7185), identifying them as actin-rich membrane protrusions (*SI Appendix*, Fig. S1 *B*−*D*). We then imaged glass-bound Alexa Fluor 594-labeled influenza A WSN (H1N1) (IAV-AF594) virions using our custom AFM-confocal setup (Fig. 1*D*). Analysis of 126 virions showed a 100% fluorescent labeling efficiency and a median virion height of 93.3 nm (*SI Appendix*, Fig. S2). These observations established the fundamental capability of virus-view AFM to detect and characterize individual virus particles with high precision.

We next set out to establish our AFM setup for IAV cell entry studies in live MDCK cells and evaluated whether virus-view AFM interferes with virus-sialic acid interactions. MDCK cells were inoculated with IAV-AF594 at 200 particles per cell and subjected to AFM imaging after 5 min. Imaging was carried out at room temperature to match the conditions which informed the ultra-narrow cantilever design (11). In the AFM image, multiple particles similar in size to those of the virus were observed along with membrane protrusions, and multiple IAV-specific fluorescent puncta were detected by confocal microscopy (Fig. 1*E*). The merged image confirmed that the three particles colocalized with the fluorescent signals, identifying them as IAV virions bound to the cell membrane surface (Fig. 1*E*). As shown in Fig. 1*F* and *SI Appendix*, Fig. S3 and Movie S2, further time-lapse imaging at 10-s intervals captured IAV-AF594 undergoing lateral diffusion on the cell surface. The trajectory of three individual IAV-AF594 particles was analyzed by single particle tracking (Fig. 1*F*), and their diffusion coefficient (D), calculated from the mean squared displacement (MSD) plots, were 0.00009 (I), 0.00067 (II), and 0.00017 (III) µm^2^/s. In logarithmic, the value of log_10_ [D (µm^2^/s)] was −4.05 (I), −3.17 (II), and −3.77 (III), respectively (Fig. 1*G*). To evaluate the potential impact of the AFM cantilever on IAV diffusion, we followed the workflow shown in *SI Appendix*, Fig. S4. MDCK cells inoculated with IAV-AF594 were first imaged with confocal microscopy alone (Step I, Fig. 1*H* and Movie S3), followed by dual imaging using virus-view AFM (Step II, Fig. 1*H* and Movie S3). We compared the cell surface diffusion coefficients of 14 individual IAV particles and observed no difference between Steps I and II (Fig. 1*I*), although the diffusion values for individual viruses fluctuated between Step I and Step II. The average value of log_10_ [D (µm^2^/s)] was −3.13 ± 0.40 (Step I) and −3.15 ± 0.37 (Step II), respectively. Two consecutive datasets acquired with confocal microscopy alone on a single virus showed the value of log_10_ [D (µm^2^/s)] of −3.03 (1st set) and −3.22 (2nd set), respectively, indicating that the diffusion coefficient of a single virus showed fluctuations even in the absence of AFM observation (*SI Appendix*, Fig. S5). These observations demonstrated that ViViD-AFM enables the detection of physiological diffusion of virions on the cell surface.

### IAV Diffusion Depends on Viral Neuraminidase Activity, Sialic Acid Density, and IAV-Subtype.

**Fig. 2.**
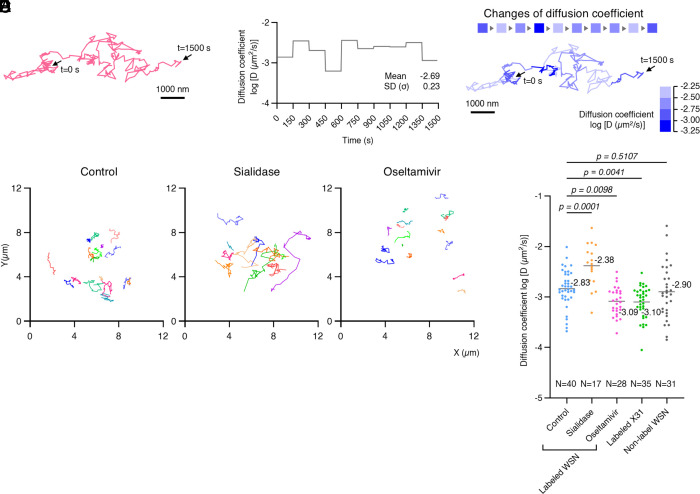
Effects of sialidase, neuraminidase, and IAV subtype on IAV cell surface diffusion. (*A*) Trajectory of a single IAV/WSN-AF594 virion, captured by time-lapse AFM imaging at 37 °C at 5-s intervals for 1,500 s. (*B*) Time course diffusion coefficient for the virion shown in panel (*A*). (*C*) Spatial variation in diffusion coefficients for the virion shown in panel (*A*): changes over time (*Top*) and corresponding trajectory (*Bottom*) were color-coded by diffusion coefficient. (*A* and *C*) (Scale bar, 1,000 nm.) (*D*) Representative trajectories ofIAV/WSN-AF594 virions at the cell surface from multiple cells under control (*Left*), sialidase-treated (*Middle*), and oseltamivir-treated (*Right*) conditions acquired by time-lapse ViViD-AFM imaging at 37 °C at 5-s intervals for 150 s. Individual trajectories are shown in different colors. (*E*) Comparison of virion diffusion coefficients under different conditions: IAV/WSN-AF594 under control (N = 40), sialidase-treated (N = 17), and oseltamivir-treated (N = 28) conditions; IAV/X31-AF594 under control conditions (N = 35); and unlabeled IAV/WSN (N = 31). Bars indicate mean values. (*B*, *C*, and *E*) Diffusion coefficient was calculated from MSD plots over 30 frames (150 s).

Lateral IAV diffusion is influenced by multivalent interactions between viral HA and sialic acid receptors (11, 12, 42–44). Consequently, the viral diffusion coefficient depends on the sialic acid density on the cell surface (42, 45). In addition, NA activity has been reported to influence IAV mobility on lipid bilayers (14). First, we investigated how a single IAV virion diffuses over time. MDCK cells were inoculated with IAV-AF594 at 200 particles per cell at 37 °C, followed by AFM imaging at 5-s intervals. A single IAV virion was tracked on the cell surface for 1,500 s (Fig. 2*A* and Movie S4). The 150-s simple moving average of log_10_ [D (µm^2^/s)] was −2.69 ± 0.23 (Fig. 2*B*). Notably, the diffusion coefficients showed microscale fluctuations, reflecting local heterogeneity in membrane properties or receptor distribution (Fig. 2 *B* and *C*).

We next interrogated the viral HA–receptor interaction via two means: by NA inhibition and enzymatic removal of sialic acids by sialidase treatment (46). NA activity to cleave sialic acid was blocked using oseltamivir (20 µM). In the control and oseltamivir-treated MDCK cells, the number of bound IAV-AF594 virions per AFM field of view was 4.1 ± 1.0 and 3.6 ± 1.1 (median ± interquartile range), respectively (*SI Appendix*, Fig. S6). Cells treated with 0.65, 2.5, 10 mUnits/mL sialidase at 37 °C for 30 min, bound 3.8 ± 1.5, 1.6 ± 0.5, and 0.04 ± 0.08 virions per AFM field of view (median ± interquartile range), respectively (*SI Appendix*, Fig. S6). For subsequent analyses, 2.5 mUnits/mL sialidase treatment was used. Using virus-view AFM, we visualized the diffusion trajectories of IAV particles and quantified their diffusion coefficients (log_10_D) (Fig. 2 *D* and *E*), which followed a Gaussian distribution (*SI Appendix*, Fig. S7). In untreated cells, the average value of log_10_ [D (µm^2^/s)] was −2.83 ± 0.37 (N = 40). In sialidase-treated cells, this value increased to −2.38 ± 0.43 (N = 17), consistent with previous in vitro reports showing that reduced sialic acid density correlates with increased viral diffusion (45) Conversely, oseltamivir treatment significantly decreased IAV mobility to −3.09 ± 0.28 (N = 28) (Fig. 2*E*). Both effects were statistically significant compared to control (sialidase: *P* = 0.0001; oseltamivir: *P* = 0.0098; Fig. 2*E*). At 15 °C, where membrane protrusion activity ceased (blue arrowheads in *SI Appendix*, Fig. S8*A*) and membrane fluidity decreased significantly, IAV diffusion was also markedly suppressed, with log_10_ [D (µm^2^/s)] dropping to −4.14 ± 0.18 (N = 30) (*SI Appendix*, Fig. S8*B*).

Additionally, we examined diffusion characteristics across different IAV strains and labeling conditions. To compare the diffusion properties of two major IAV subtypes that commonly infect humans, we analyzed both H1N1 and H3N2 strains. While the diffusion coefficient (log_10_D) of IAV/WSN (H1N1) was −2.83 ± 0.37 (N = 40), the IAV/X31 (H3N2) strain exhibited significantly lower mobility with a value of −3.10 ± 0.32 (N = 35) (Fig. 2*E*). Notably, the high-resolution imaging capability of virus-view AFM enabled tracking of unlabeled IAV/WSN particles, which displayed a diffusion coefficient of −2.90 ± 0.54 (N = 31) (Fig. 2*E*). No significant difference was observed between the diffusion coefficients of labeled and unlabeled viruses (*P* = 0.51), validating that fluorescent labeling does not affect the viral mobility on the cell surface.

### IAV Cell Surface Diffusion Reduces with the Onset of Clathrin Coat Assembly.

**Fig. 3.**
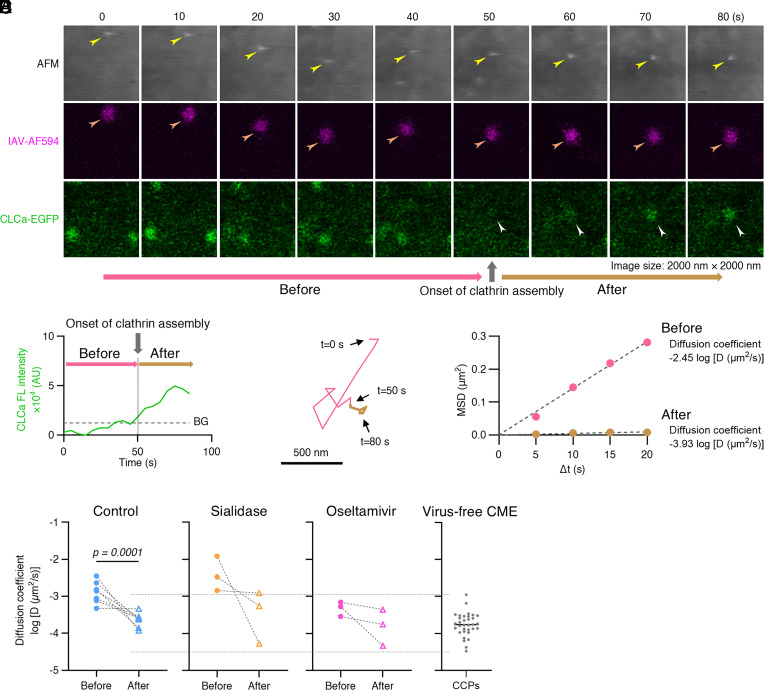
IAV cell surface diffusion before and after clathrin assembly. MDCK cells expressing clathrin light chain a (CLCa) fused with EGFP (CLCa-EGFP) were inoculated with IAV/WSN-AF594 at 37 °C and imaged using ViViD-AFM at 5-s intervals. (*A*) Time-lapse detection of a diffusing IAV/WSN virion (yellow and orange arrowheads) and clathrin assembly (white arrowheads). Image size: 2,000 × 2,000 nm^2^. AFM height range: 400 nm. (*B*−*D*) Analysis of cell surface IAV diffusion for the virion shown in panel (*A*). (*B*) CLCa fluorescence (FL) intensity at virion attachment sites over time. Clathrin assembly onset was defined as the time when CLCa FL intensity exceeded the background level (BG) indicated by a dashed line. AU, arbitrary units. (*C*) Trajectory of IAV/WSN-AF594 virion on the cell surface, color-coded to show movement before (pink) and after (brown) onset of clathrin assembly. (Scale bar, 500 nm.) (*D*) Analysis of single virion diffusion coefficient before and after onset of clathrin assembly. Diffusion coefficient (*D*) is indicated on the graph. MSD, mean squared displacement. (*C* and *D*) The trajectory of IAV and the analysis of diffusion coefficient were obtained based on fluorescence data. (*E*) Diffusion coefficients of individual virions before and after clathrin assembly under control (N = 7), sialidase-treated (N = 3), and oseltamivir-treated (N = 3) conditions, compared with virus-free CCPs (mean = −3.76, N = 33). Dashed lines connect the measurements of individual virions.

Lateral diffusion on the cell surface allows IAV virions to accumulate in membrane regions enriched with sialylated receptors, enabling multivalent engagement. This preferential localization to receptor-rich domains, particularly areas with high EGFR density as demonstrated by Sieben et al. (39) facilitates receptor clustering that in turn activates signaling pathways which initiate clathrin-dependent or -independent endocytosis (16, 21). To investigate the relationship between IAV diffusion and clathrin recruitment, we inoculated MDCK cells stably expressing clathrin light chain a (CLCa)-EGFP with IAV/WSN-AF594 (200 particles per cell) at 37 °C, followed by ViViD-AFM imaging at 5-s intervals (Fig. 3*A*). In this representative example, de novo clathrin coat assembly underneath the virion was first detected 50 s after imaging began (Fig. 3 *A* and *B* and Movie S5). The diffusion coefficient of the IAV particle, calculated from the MSD plot, decreased from log_10_ [D (µm^2^/s)] = −2.45 before clathrin recruitment to −3.93 afterward (Fig. 3 *C* and *D*). Notably, the reduction in the viral mobility following clathrin coat assembly was also observed upon sialidase and oseltamivir treatments (Fig. 3*E*). The diffusion coefficient of IAV after clathrin recruitment was within the range of diffusion values observed for clathrin-coated pits (CCPs) formed in the absence of virus in all conditions (Fig. 3*E* and *SI Appendix*, Fig. S9); these results support the notion that virions become confined within assembling CCPs.

### Membrane Bulges Cover Virus Particles During IAV Endocytosis.

**Fig. 4.**
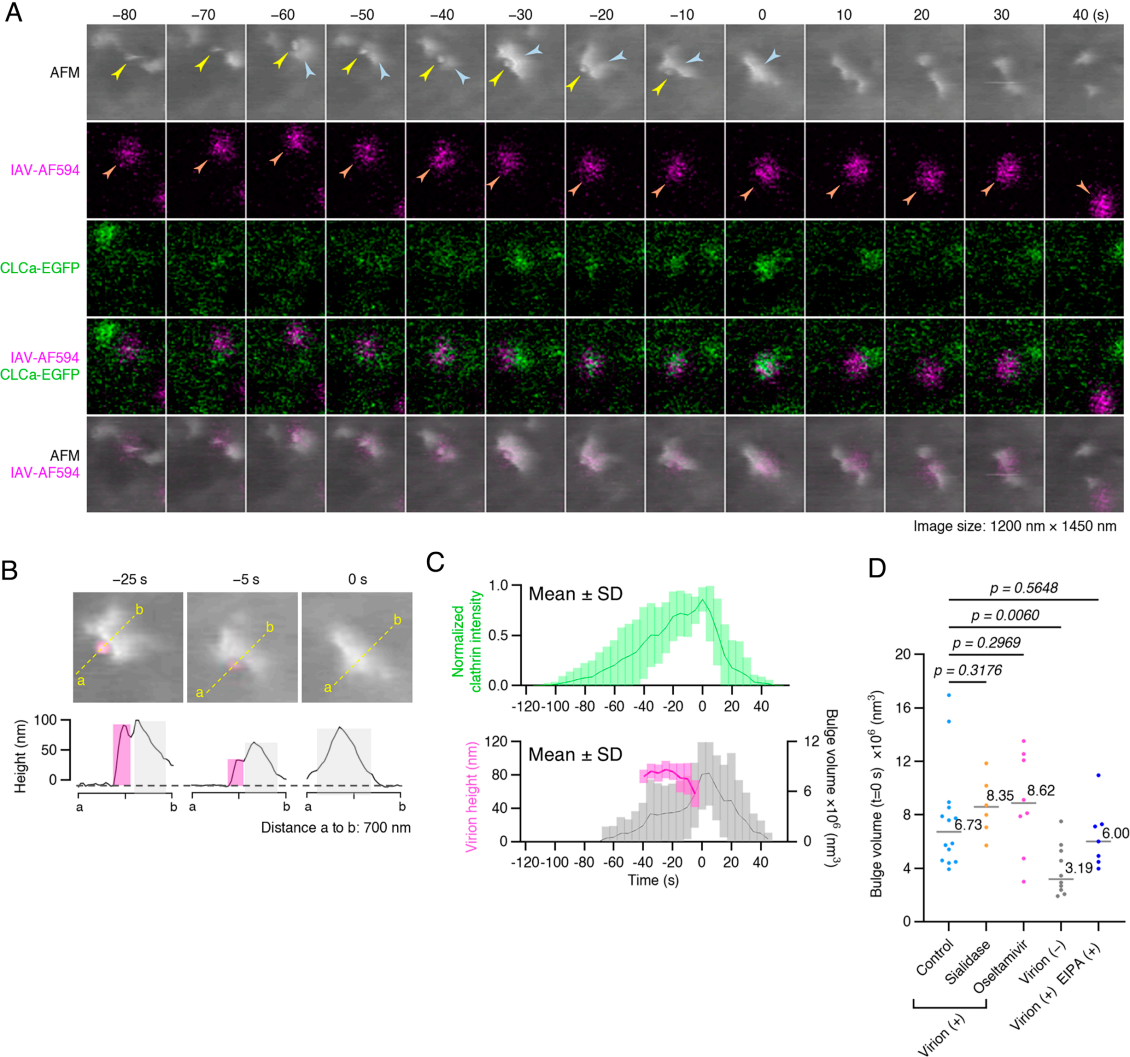
Membrane bulges cover virus particles during IAV CME. MDCK cells expressing CLCa-EGFP were inoculated with IAV/WSN-AF594 and imaged using ViViD-AFM at 37 °C at 5-s intervals. (*A*) IAV CME captured by time-lapse AFM imaging at 37 °C at 5-s intervals. Sequential images shown at 10-s intervals from *Top* to *Bottom*: AFM, IAV-AF594 (magenta), CLCa-EGFP (green), merge of IAV-AF594 and CLCa-EGFP, and merge of AFM and IAV-AF594. Arrowheads indicate virion morphology (yellow), membrane bulges (blue), and virion fluorescence (orange). Virion morphology disappeared at t = 0 s. Image size: 1,200 × 1,450 nm^2^. AFM height range: 400 nm. (*B*) Cross-sectional profiles (*Bottom*) along dashed lines over a virion in AFM images (*Top*) from panel (*A*) at −25, −5, and 0 s. Virions and membrane bulges are shown with magenta and gray, respectively. Virion morphology in the AFM image is colored with magenta. The gray dashed line indicates the membrane baseline (height = 0 nm). (*C*) Quantification of clathrin intensity (*Top*, green) (N = 11), virion height (bottom, pink) (N = 8) and bulge size (*Bottom*, gray) (N = 11) during IAV endocytosis. Data are shown as mean ± SD. (*D*) Comparison of membrane bulge volumes during CME: IAV (+) CME under control (N = 14), sialidase-treated (N = 6), and oseltamivir-treated (N = 8) conditions; IAV-free CME (N = 10); and IAV (+) CME under EIPA-treatment (N = 7). Bars indicate mean values.

Next, we analyzed IAV internalization via CME, the major cell uptake pathway for the virus (16, 21). MDCK cells stably expressing CLCa-EGFP were inoculated with IAV/WSN-AF594 (200 particles per cell) at 37 °C, followed by ViViD-AFM imaging at 5-s intervals (Fig. 4*A*). The time point at which the virion disappeared from AFM view but was still detectable by fluorescence was defined as t = 0 s (Fig. 4 *A* and *B* and Movie S6). At the moment of clathrin-mediated IAV endocytosis, a membrane bulge with a height of ~100 nm was observed enveloping the IAV virion at the cell surface. Similar membrane bulges were also detected during CME events in the absence of virus, covering and closing the CCPs from one side (*SI Appendix*, Fig. S9), as previously reported (24, 25). The internalized IAV then underwent rapid, directional movement toward the perinuclear region (*SI Appendix*, Fig. S10 *A* and *B* and Movie S7), confirming intracellular trafficking following bulge-promoted endocytosis.

As the clathrin coat grew, membrane bulges began to form (Fig. 4 *A*−*C* and *SI Appendix*, Fig. S11). Beginning at t = −25 s, the IAV virion started to sink into the membrane, with its mean height above the membrane baseline decreasing from approximately 88 nm (Fig. 4 *B* and *C* and *SI Appendix*, Fig. S11*B*). During the IAV sinking process, the average membrane bulge volume increased from 3.4 × 10^6^ to 8.1 × 10^6^ nm^3^, reaching heights over 100 nm above the membrane baseline (Fig. 4 *B* and *C*). IAV-containing CCP closure occurred approximately 59 nm above the cell membrane baseline (Fig. 4*C*). Both the clathrin signal intensity and the membrane bulge volume peaked at t = 0 s in CME, regardless of viral presence (Fig. 4*C* and *SI Appendix*, Fig. S12). The presence of IAV significantly increased the membrane bulge size, with average volumes of 6.73 × 10^6^ nm^3^ and 3.19 × 10^6^ nm^3^ for IAV-positive (N = 14) versus IAV-free CME (N = 10), respectively (*P* = 0.006) (Fig. 4*D*). Neither sialidase nor oseltamivir treatment affected the membrane bulge size during IAV CME (Fig. 4*D*). These membrane bulges strongly correlated with EGFP-Lifeact fluorescence, indicating their actin-rich nature (*SI Appendix*, Fig. S13).

To distinguish virus-induced CME bulges from macropinocytosis-related membrane protrusions, we treated cells with the macropinocytosis inhibitor 5-(N-ethyl-N-isopropyl)-Amiloride (EIPA). While EIPA treatment significantly reduced membrane ruffling (41) (*SI Appendix*, Fig. S14*A* and Movie S8), membrane bulging associated with virus-induced CME remained unchanged (Fig. 4*D* and *SI Appendix*, Fig. S14 *B* and *C* and Movie S9).

## Discussion

In this study, we enhanced the minimally invasive capabilities of AFM by introducing an ultra-narrow cantilever that preserves the interaction between IAV virions and the cell membrane. Utilizing live-cell correlative imaging of nanoscale morphology and fluorescence with ViViD-AFM, we thoroughly analyzed the early stages of IAV host cell entry. Virus-view AFM revealed the diffusion characteristics of IAV virions preceding CME, the factors influencing IAV diffusion, and the actin-regulated cell membrane bulging dynamics associated with IAV internalization by CME.

### Minimally Invasive Performance of AFM.

Ando et al. defined that the force impulse exerted on a sample during AFM imaging is determined by the product of peak force and contact time (47). Thus, the force impulse can be minimized by either reducing peak force or shortening contact time.

The high-speed AFM developed in 2001 exemplified the latter approach (29). Enhancing the cantilever resonance and scanning frequency significantly shortened the contact time, enabling video-rate resolution of dynamic molecular events.

For cell-imaging AFM, however, it is difficult to reduce the contact time by increasing scanning frequency. This is because a trade-off exists between the AFM scan area and scanning frequency, which limits the scan frequency in cell imaging applications that require µm-scale observations. Therefore, we chose to reduce the peak force while maintaining a constant scanning frequency. This approach led to the design of an ultra-narrow cantilever (Fig. 1*B*). Its minimized width is optimized for the optical lever sensor’s detection limits and enables low-invasive cell imaging.

To evaluate whether virus-view AFM perturbs the virus-cell interaction, we compared consecutive diffusion datasets from a single virus and observed fluctuations in diffusion coefficients before and during AFM scanning (Fig. 1*I*). Similar fluctuations in diffusion coefficients were also observed between consecutive fluorescence-only datasets (*SI Appendix*, Fig. S5). This suggests that these fluctuations stem from differences in the local environment that influence viral mobility (discussed later in detail), rather than from mechanical perturbation caused by the AFM cantilever.

Virus-view AFM, equipped with an ultra-narrow cantilever, has the broad potential to visualize virus–cell interactions for various viruses while preserving native binding dynamics. Compared to other viruses, IAV interactions with the cell membrane are particularly weak due to the high HA-sialic acid dissociation constant (9, 48, 49). Thus, the capability of our custom AFM setup to successfully image IAV cell entry highlights its broader applicability for studying the cell entry of other viruses, including those with stronger receptor affinities.

### Early Stages of IAV Host Cell Entry.

The lateral diffusion of IAV virions on the cell surface is an essential process for engaging sufficient cell surface receptors and initiating signal transduction. The cell membrane contains regions where sialic acid receptors, including EGFR, are highly concentrated, creating a heterogeneous distribution of sialic acid density across the cell membrane (39). Our findings demonstrate that virion diffusion coefficients correlate inversely with sialic acid content: Virions move more freely (with higher diffusion coefficients) in regions with decreased sialic acid density (Fig. 2 *D* and *E*). Accordingly, virions diffusing across the membrane exhibit fluctuating diffusion coefficients that reflect spatial variation in receptor density (Fig. 2 *B* and *C* and *SI Appendix*, Fig. S5). Sieben et al. (39) showed that these virions migrate toward and become preferentially trapped in microdomains rich in sialylated receptors. This directed movement enhances multivalent binding which stabilizes the virion at these sites (Fig. 3 and *SI Appendix*, Fig. S15*A*). Once IAV particles localize in these receptor-rich domains, they trigger receptor clustering and activate downstream signaling pathways leading to clathrin accumulation and endocytosis (16).

While most virions bound to the cell membrane trigger clathrin assembly within 2 to 3 min of attachment (16), our experiments revealed that some IAV particles continued to diffuse on the cell surface for as long as 1,500 s (Fig. 2 *B* and *C*). Previous studies have shown that efficient virion uptake requires a finely tuned balance between viral HA–receptor binding and NA activity (12, 44, 50–52). Notably, virions that diffused for extended periods exhibited mean diffusion coefficients and variations within the normal distribution shown in Fig. 2*E* (Control), suggesting these virions likely had a typical HA/NA balance. Therefore, we hypothesize that virions with extended mobility simply failed to encounter receptor clusters for productive internalization.

Our study demonstrates that virions diffusing on the cell surface exhibit decreased diffusion coefficients when NA activity is blocked (Fig. 2 *D* and *E*). At physiological temperature (37 °C), oseltamivir treatment caused a relatively modest effect on IAV diffusion (Fig. 2*E*) compared to previous reports (14). However, under reduced membrane fluidity conditions (15 °C), oseltamivir significantly decreased virion mobility in line with these earlier findings (*SI Appendix*, Fig. S8). This observation suggests that the attenuated effect of NA inhibition at physiological temperature likely results from the counteracting influence of increased membrane fluidity at 37 °C, which partially overcomes the mobility restrictions typically associated with oseltamivir treatment.

Our AFM diffusion analysis successfully distinguished between IAV subtypes [IAV/X31 (H3N2) and IAV/WSN (H1N1)] with statistical significance (Fig. 2*E*). This capability is valuable given the extensive subtype diversity among IAVs which comprise at least 18 HA and 11 NA (2), contributing to broad host range and pathogenicity. While conventional microscopy lacks the resolution and sensitivity to quantify subtype-specific viral mobility at the cell surface, virus-view AFM can provide critical information on diffusion coefficients that reflect subtype-dependent virus–membrane interactions.

To analyze the diffusion process of virions in greater detail, higher temporal resolution in imaging is essential. In this study, due to performance constraints of our AFM system, images were acquired at 5-s intervals. To calculate diffusion coefficients as reliably as possible, analysis was conducted using 5 to 30 data points (25 to 150 s). As a result, we could not temporally resolve the diffusion process such as transient directional changes or stalling events with sufficient precision. To more accurately characterize the temporal relationship between virion behavior and clathrin recruitment, we believe that future ViViD-AFM systems will require at least an order of magnitude improvement in time resolution.

Observations using virus-view AFM also revealed that an actin-rich membrane bulge promoted CCP closure during IAV-induced CME, which typically occurred ca. 60-nm above the cell membrane baseline (Fig. 4 *B* and *C* and *SI Appendix*, Fig. S11). While vesicular stomatitis virus (VSV) (~200 nm in length) is considered too large to fit within a CCP and thus requires actin assembly during CME (53), IAV (~90 nm diameter, *SI Appendix*, Fig. S2) matches CCP dimensions. Nevertheless, our findings demonstrate that IAV uptake required a significantly larger membrane bulge than virus-free CME events (Fig. 4*D*), with actin-rich membrane bulges having volumes approximately twice that of regular CME. These bulges were not affected by EIPA treatment (Fig. 4*D*), suggesting a shared underlying mechanism with CIP4-dependent membrane deformation reported in nonviral CME (24, 25). After engaging with sialic acid clusters and reduced diffusion, IAV primes the cell for entry by activating cell signaling pathways, which involve receptor tyrosine kinases (54). This triggers the assembly of a clathrin coat around the virion (16) and the formation of actin-rich membrane bulges that envelop it as it sinks into the CCP (*SI Appendix*, Fig. S15*B*). Following dynamin-mediated membrane fission and the formation of a clathrin-coated vesicle, the internalized virion is transported to early endosomes and then toward the nucleus along microtubules (*SI Appendix*, Fig. S10) (15, 55).

### Summary.

Our virus-view AFM imaging system has the potential to significantly advance the understanding of the mechanobiology underlying pathogen-cell membrane interactions. It allows high-resolution, live-cell visualization of viral uptake, providing direct insight into the dynamic processes of endocytosis. By simultaneously observing the localization of endocytosis-related factors, identification of clathrin-independent uptake pathways becomes possible. In the future, virus-view AFM could be applied to study various biological activities occurring at the cell surface of mammalian cells or bacteria, such as the formation and uptake of spherical and filamentous IAVs and extracellular vesicles (EVs), and the uptake of lipid nanoparticles (LNPs) (56, 57). Notably, EVs and LNPs are used in drug delivery to enhance therapeutic efficacy (56, 57), and a critical aspect of EV drug delivery is its cellular uptake, with EVs diffusing on cell surfaces (58) and undergoing endocytosis (59). Our live, dual-mode imaging approach presents a unique opportunity to study these diffusion and entry processes in real-time. Overall, ViViD-AFM is a versatile tool with the potential to transform future discoveries in membrane biology, virus–host interactions, and drug discovery research.

## Materials and Methods

### Preparation of Viruses.

Influenza A/WSN/33 (H1N1) and A/X31 (H3N2) virus was prepared as previously described (55). Briefly, the virus was propagated in 30 chicken eggs at 35 °C for 2 d. After 1 d of incubation at 4 °C, the allantoic fluid was collected and clarified by centrifugation at 10,000 rpm for 20 min. The clarified fluid was then concentrated by centrifugation at 100,000×*g* for 90 min. Further purification involved two cycles of centrifugation using Iodixanol (Sigma-Aldrich, St. Louis, MO) gradient ranging from 10 to 40% at 100,000×*g* for 90 min. Viral bands were collected and resuspended in NTC buffer (100 mM NaCl and 20 mM Tris-HCl, pH 7.4, 5 mM CaCl_2_). The viral titer, plaque-forming units (pfu) per mL, was determined in MDCK cells. Aliquots of the virus were stored in NTC buffer at −80 °C until use.

### Fluorescent Labeling of Viruses.

Influenza A/WSN or A/X31 virus was labeled with Alexa Fluor 594-NHS ester dye (A37572, Thermo Fisher, Carlsbad, CA) as follows. NaHCO_3_ (pH ≈ 8) was added to the virus solution to a final concentration of 0.14 M. Alexa Fluor 594 ester dye, suspended in DMSO, was then added to obtain the final dye concentration of 50 µM. The virus, at a concentration of 0.8 × 10^9^ pfu/mL, was incubated with dye for 1 h in the dark at room temperature. To remove unbound dye, the reaction mixture was loaded onto a PD MiniTrap^™^ G-25 column (28918007, GE Healthcare, Little Chalfont, UK) pre-equilibrated with HBS buffer. The influenza A virus conjugated with Alexa Fluor 594 (IAV-AF594) was then eluted from the column with HBS buffer. Note that it is unclear why the virus particles were highly labeled with Alexa Fluor 594-NHS ester dye in the presence of high concentration of Tris (~2 mM), which typically competes for NHS reactions.

### Plasmids.

The cDNA for CLCa was amplified from eGFP-CLCa (a kind gift from Dr. Asuka Nanbo, Nagasaki University) (60) by PCR with the primers, CLCa forward (GGCTCGAGATGGCTGAGCTGGATCC) and CLCa reverse (TAGCGGCCGCTCAGTGCACCA GCGGG). The amplified sequence was then subcloned into the XhoI/NotI sites of the pCX4puro-EGFP vector (a kind gift from Dr. Kazuhiro Aoki, National Institute for Basic Biology). For Lifeact expression, pFX-Lifeact-EGFP or pFX-Lifeact-mCherry was used (61).

### Cell Culture.

MDCK cells (CRL-34, American Type Culture Collection, Manassas, VA) were cultured under a 5% CO_2_ humidified atmosphere at 37 °C in Dulbecco’s Modified Eagle’s Medium (DMEM, Sigma-Aldrich) supplemented with 10% fetal bovine serum (Thermo Fisher Scientific). For transient expression of Lifeact-EGFP or Lifeact-mCherry, the pFX-Lifeact-EGFP or pFX-Lifeact-mCherry expression vector was transfected into MDCK cells using Polyethylenimine Max (Polysciences, Warrington, PA). To establish cell lines stably expressing CLCa-EGFP, expression vectors for CLCa fused with EGFP were linearized by ScaI and introduced into the MDCK cells using nucleofection according to the manufacturer’s protocols (Amaxa Biosystems, Cologne, Germany). 1 d after transfection, cells were cultured in DMEM containing 4 µg/mL Puromycin (Wako, Kyoto, Japan). Resistant clones were collectively isolated 2 d after transfection. Stably expressing cells were maintained in DMEM supplemented with 10% FBS.

### ViViD-AFM System.

The virus-view AFM composed of a tip-scan type AFM system (BIXAM™; Olympus Corporation, Tokyo, Japan) equipped with an ultra-narrow cantilever and a multicolor confocal laser scanning microscope system (FV1200, Olympus Corporation). The AFM head used in this study is based on a design first developed in 2013 (37), with improvements made in 2018 (24). In the original configuration, the objective lens of the optical beam deflection (OBD) sensor was mounted on an XY movable stage, allowing it to move synchronously with the cantilever and maintain alignment of the laser spot. The 640 nm laser beam was directed through a collimator lens, polarized beam splitter, quarter-wave plate, objective lens, and a transparent glass window to focus on a narrow, 2 µm-wide cantilever. Reflected light followed a reverse optical path and was detected by a split photodiode. Laser positioning was monitored using an optical microscope and optimized by adjusting the laser diode position to maximize the reflected intensity. In 2018, improvements included the expansion of the scanning range and replacement of the 640 nm laser diode with an 880 nm superluminescent diode as the light source for the OBD sensor. AFM Scanning System Software Version 2.0.2.0 (Olympus Corporation) was used for acquiring time-lapse AFM images and for morphological analysis. The AFM used a tapping mode with phase feed-back control, scanning at a frequency of 30 Hz or 60 Hz in the X direction to image one frame in 10 or 5 s, respectively. 320 data points were sampled in the X direction and 240 in the Y direction. AFM images of cells were displayed in a maximum 6 × 4.5 × 400 nm (X × Y × Z) area with a resolution of 320 × 240 (X × Y) and 256 shades of an 8-bit gray scale (Z) where bright areas are higher and dark areas are lower. An 880 nm SLD (Hamamatsu Photonics, Japan) was used for the cantilever optical beam deflection (OBD) sensor. A customized ultra-narrow cantilever (USC-F0.8-k0.05; NanoWorld AG, Neuchâtel, Switzerland) with an electron beam deposited (EBD) tip < 10 nm radius of curvature at the free end was used. The multicolor confocal laser scanning microscope (FV1200, Olympus Corporation) consisted of an electric inverted fluorescence microscope IX83, two lasers (473 and 559 nm), and two fluorescence channels with High sensitivity GaAsp Detectors. Time-lapse fluorescence images in 12.3 × 12.3 µm (X × Y) area with a resolution of 512 × 512 (X × Y) were obtained using a 100 × objective under oil emersion, with a 2 μs/pixel scan speed and 10 or 5 s interval. For correlative imaging, the targeted cell was selected using a phase contrast microscope with a 20 × or 60 × objective lens. Using fluorescence observation with the confocal microscope and 100 × objective lens, the target area of the cell was placed at the center of the fluorescence image. The AFM probe with autofluorescence was then approached onto the target area, and AFM imaging was initiated. Sequential images captured by AFM and confocal microscopy were overlaid by using AviUTL (http://spring-fragrance.mints.ne.jp/aviutl/), based on the AFM tip position as previously described (24). A montage of AFM and fluorescence images was trimmed from the stack images of 6.0 × 4.5 or the 12.3 × 12.3 μm image using the ImageJ software Version 2.14.0/1.54f (National Institutes of Health; https://imagej.nih.gov/ij/). The virus-view AFM system was set up in a BSL-2 laboratory, where all experiments were subsequently conducted.

### Phase Control Adjustment Method.

Our AFM system oscillated the cantilever at a fixed frequency of around 300 kHz and used feedback control based on Acos(φ +Ψ) (Patent No.: US 9,453,856 B2, US.9335341.B2, US.9977049.B2). Here, φ represents the phase shift amount when the probe contacts the sample, Ψ is the preset phase offset amount, and A is the vibration amplitude of the cantilever. We used an NF Corporation phase detector module (custom made). The phase offset Ψ can be adjusted within - π /2 ≤ Ψ ≤π /2. We typically set it within the range - π /4 ≤ Ψ ≤π /4, and in this study, we performed the observation with Ψ≃π/4. Our AFM system can monitor the values of Acos(φ +Ψ) and Asin(φ +Ψ), and we adjusted the phase offset so that the values of Acos(φ +Ψ) and Asin(φ +Ψ) match, when φ = 0, i.e., Ψ≃π/4.

### Peak Force Estimation of the ViViD-AFM System.

The peak force generated by periodic contact between the probe and sample includes both dynamic (AC) and static (DC) force components. Meanwhile, the multivalent binding force between HA and sialic acid (10 to 25 pN) measured in previous studies (39) is a static binding force, and the dynamic force characteristics remain unknown.

Therefore, in this study, to compare peak force and multivalent binding force in the same dimension, we quantified the static component of the peak force and compared it with 10pN.

Peak force reduces the oscillation amplitude of the cantilever through its dynamic component and causes cantilever deflection (bending) through its static component. The relationship between the reduction in cantilever oscillation amplitude (0 to Peak) ΔA (nm) and cantilever deflection δ (nm) is given by

[3] δ≈ΔA/Q,

where Q is the mechanical quality factor of the cantilever in liquid. For our ultra-narrow cantilever, Q≦2. We calculated using Q=2.

The static force (Fs) of the peak force, with the cantilever spring constant denoted as k (N/m), is determined by

[4] Fs=δ∙k≈ΔA∙k/Q.

Based on Eq. **4**, we determined ΔA and k such that ΔA∙k/Q (nN) ≦ 0.01 (nN).

We designed a cantilever shape with a spring constant of 0.04 (N/m) and set control parameters to achieve an oscillation amplitude reduction of ΔA = 0.5 (nm). This reduction of 0.5 (nm) corresponds to 10% of the cantilever’s free amplitude (0 to Peak).

### Live AFM Imaging of Viruses at the Cell Surface.

For correlative imaging, glass slides (KB218-0A; Toa optical technologies, LTD, Tokyo, Japan), featuring a circular 15 mm diameter cover glass on a water-repellent printed glass slide were used. MDCK cells were seeded on poly-L-lysine-coated (P4707; Sigma-Aldrich) glass slides 1 d before imaging. Prior to microscopic observation, the medium was replaced with phenol red-free DMEM/F12 (11039-021; Thermo Fisher Scientific) and the cells were inoculated with IAV-AF594 at a multiplicity of infection (MOI) of 100. The number of virus particles per cell was determined using correlative AFM-fluorescence imaging in representative fields (6 × 4.5 µm^2^). Virus particles were counted when they satisfied both criteria: 1) positive for Alexa Fluor 594 fluorescence and 2) detectable by AFM as discrete particles with heights consistent with influenza virions (~90 nm). Multiple representative fields were analyzed to calculate the average particle density per unit area, and the total number of particles per cell was estimated by multiplying this density by the average cell surface area. This quantification revealed that MOI 100 conditions corresponded to approximately 200 particles per cell. AFM imaging was initiated 5 min after inoculation at 15 °C, room temperature (27 °C), or 37 °C. The cell periphery was selected as the imaging area. For neuraminidase inhibition, cells and viruses were treated with 20 µM oseltamivir carboxylate (GS-4021; Cayman, Ann Arbor, MI) 30 min prior to virus inoculation. To reduce sialic acid density on the cell membrane, cells were treated with 0.625, 2.5, or 10 mUnit/mL sialidase (N7885; Sigma) for 30 min at 37 °C and were washed twice with DMEM/F12 medium before virus inoculation. For macropinocytosis inhibition, cells were treated with 80 µM 5-(N-ethyl-N-isopropyl)-Amiloride (EIPA) for 30 min prior to virus inoculation. All the experiments were conducted in a BSL-2 laboratory using live viruses.

### AFM Imaging of Viruses Immobilized on a Glass Substrate.

To analyze virus morphology and fluorescence labeling efficiency, IAV-AF594 was diluted approximately 200-fold with PBS. The virus was then immobilized onto a glass surface precoated with 1.0 mg/mL fetuin (F3004; Sigma-Aldrich) as described previously (14). The immobilized viruses were observed by ViViD-AFM at room temperature.

### Image Analysis.

To track and analyze IAV cell surface diffusion, virus trajectories were generated with the use of the Multidimensional Image Analysis module of the MetaMorph software (Molecular Devices, San Jose, CA). From fluorescence stack files, fluorescence spots of IAV-AF594 were segmented with adaptive thresholding and by pairing spots in each frame according to proximity and similarity in intensity. The X-Y-coordinates of IAV over time were exported as an excel file. Mean square displacement (MSD) of virus was plotted over time and the diffusion coefficient (D) was calculated by linearly fitting the MSD curve based on the equation below (62), representing two-dimensional free diffusion.

[5] $$MSD\left(\Delta t\right)=4D∙\Delta t.$$

To obtain the fluorescence intensity profile of EGFP-Lifeact or IAV-AF594, the plot profile tool of ImageJ software was used. The cross-section profile of IAV virion and membrane was obtained using AFM Scanning System Software Version 2.0.2.0 (Olympus). From cross-section profiles, pit diameter and virion height were obtained by measuring the distance between 2 points at the edge of the invagination on the section profile as previously described (24) and measuring the distance between the membrane base line and the peak height of virion.

CLCa fluorescence intensity was quantified with ImageJ by measuring total fluorescence intensity within a 300 nm diameter circle centered at the virion’s center of gravity and subtracting the background from a 1,500 nm diameter circle. A moving average plot (2 points) of CLCa fluorescence intensity over time was generated, and the time when CLCa intensity exceeded the background level was defined as the onset of clathrin assembly.

Membrane protrusion volume was measured with ImageJ. On background-subtracted images (using the rolling ball tool), the volume of membrane protrusions was measured within a 500 nm diameter circle centered at the virion’s center of gravity.

### Statistical Analysis.

Data are presented as mean ± SD (unless indicated otherwise) and were compared between two conditions with Student’s *t* test or among more than two conditions by Dunnett’s test. The Wilcoxon signed-rank test was applied for comparison between paired samples. *P*-values are indicated in the respective figure panels. All statistical analyses were performed using Prism 10 Version 10.2.3 (GraphPad Software, Boston, MA).

## Supplementary Material

Appendix 01 (PDF)

Movie S1. Morphological imaging of the live MDCK cell surface. Time-lapse AFM imaging showing representative membrane morphological dynamics at 27°C at 10-s intervals (related to Fig. 1C and SI Appendix, Fig. S1A). The topographic AFM image displays height variations up to 400 nm, where brighter areas indicate higher regions and darker areas indicate lower regions. Image size: 6.0 × 4.5 μm^2^.

Movie S2. IAV lateral diffusion on the live MDCK cell surface. Time-lapse ViViD-AFM imaging showing representative IAV-AF594 virions diffusing on the cell membrane (related to Fig. 1E–F and SI Appendix, Fig. S3). Sequential images were acquired at 10-s intervals at 27°C. The movie shows the morphology of the cell surface virion (left), IAV-AF594 fluorescence (middle), and the merged image of morphology and fluorescence (right). Image size: 6.0 × 4.5 μm^2^. AFM height range: 400 nm. Arrowheads indicate three virions on the cell membrane.

Movie S3. Assessment of the effect of AFM imaging on IAV cell surface diffusion. Time-lapse imaging showing representative IAV cell surface diffusion in two steps (related to Fig. 1H and I), confocal imaging alone (step I) followed by ViViD-AFM imaging (Step II). Sequential images were acquired at 10-s intervals at 27°C. The movie shows IAV-AF594 fluorescence (left), morphology of cell surface virion (middle), and the merge of morphology and fluorescence (right). Image size: 6.0 × 4.5 μm^2^. AFM height range: 400 nm. Arrowheads indicate two virions on the cell membrane.

Movie S4. IAV cell surface diffusion on live MDCK cell. Time-lapse AFM imaging showing representative IAV virion diffusion on the cell membrane from t=225 s to 575 s (related to Fig. 2A–C). Sequential images were acquired at 5-s intervals at 37°C. Arrowhead indicates the tracked virion. AFM image: 6.0 × 4.5 μm^2^. AFM height range: 400 nm.

Movie S5. IAV cell surface diffusion on live MDCK cell before and after clathrin assembly. Time-lapse ViViD-AFM imaging (t=0 s to 80 s) showing IAV virion diffusion on the cell membrane before and after the onset of clathrin assembly at t=50 s (related to Fig. 3A–D). Sequential images were acquired at 5-s intervals at 37°C, showing: morphology of virion and cell membrane (left), IAV-AF594 fluorescence (magenta, middle), and CLCa-EGFP fluorescence (green, right). Arrowheads indicate the virion position in AFM images, IAV-AF594 fluorescence, and the site of clathrin assembly. Image size: 6.0 × 4.5 μm^2^. AFM height range: 400 nm. Clathrin assembly at the arrowhead position is observed from t=50 s.

Movie S6. Moment of IAV internalization into an MDCK cell. Time-lapse AFM three-dimensional movie showing a representative IAV virion internalization event (related to Fig. 4A and B). Sequential images were acquired at 5-s intervals at 37°C. The virion was colored in magenta.

Movie S7. Tracking of an IAV virion on an MDCK cell before and after CME. Time-lapse ViViD-AFM imaging showing the virion trajectory from t=-230 s to 180 s, with internalization occurring at t=0 s (related to SI Appendix, Fig. S8). Sequential images were acquired at 5-s intervals at 37°C, showing the merged fluorescence of CLCa -EGFP (green) and IAV-AF594 (magenta) (left), AFM morphology of virion and cell membrane (middle) and merged fluorescence and morphological data (right). Arrowhead indicates the virion morphology visible by AFM. In fluorescence images, the tracked virion trajectory is overlaid (orange: extracellular, gray: intracellular). Image size: 6.0 × 8.6 μm^2^ (fluorescence, left) and 6.0 × 4.5 μm^2^ (AFM and merged images, middle and right). AFM height range: 400 nm.

Movie S8. Effect of EIPA treatment on the live MDCK cell surface morphology (related to SI Appendix, Fig. S12A). Time-lapse AFM imaging showing dynamics of the MDCK cell surface morphology before and after 20-min EIPA treatment at 37°C. Sequential images were acquired at 5-s intervals at 37°C. Image size: 6.0 × 4.5 μm^2^. AFM height range: 400 nm.

Movie S9. IAV CME in an EIPA-treated MDCK cell (related to SI Appendix, Fig. S12B). Time-lapse AFM imaging showing representative membrane morphological changes during IAV CME, with internalization occurring at t=0 s. Sequential images were acquired at 5-s intervals at 37°C. The virion was colored in magenta. Scale bar: 1000 nm. AFM height range: 400 nm.

## Data Availability

Dataset data have been deposited in Zenodo (10.5281/zenodo.14631321) (63).
