# Supplementary material for: Enhanced visualization of influenza A virus entry into living cells using virus-view atomic force microscopy
Source: Proc Natl Acad Sci U S A. 2025 Sep 18;122(38):e2500660122. doi: 10.1073/pnas.2500660122 (PMC12478160; doi:10.1073/pnas.2500660122)
Supplement: Supplementary file 1 — Appendix 01 (PDF) [file pnas.2500660122.sapp.pdf]

PNAS

**Supporting Information for**

# Enhanced Visualization of Influenza A Virus Entry in Living Cells Using Virus-View Atomic Force Microscopy

Aiko Yoshida, Yoshitsugu Uekusa, Takeshi Suzuki, Michael Bauer, Nobuaki Sakai, and Yohei Yamauchi

Correspondence: Nobuaki Sakai and Yohei Yamauchi
Email: bixam.sakai@gmail.com, yohei.yamauchi@pharma.ethz.ch

**This PDF file includes:**

Figures S1 to S15
Legends for Movies S1 to S9

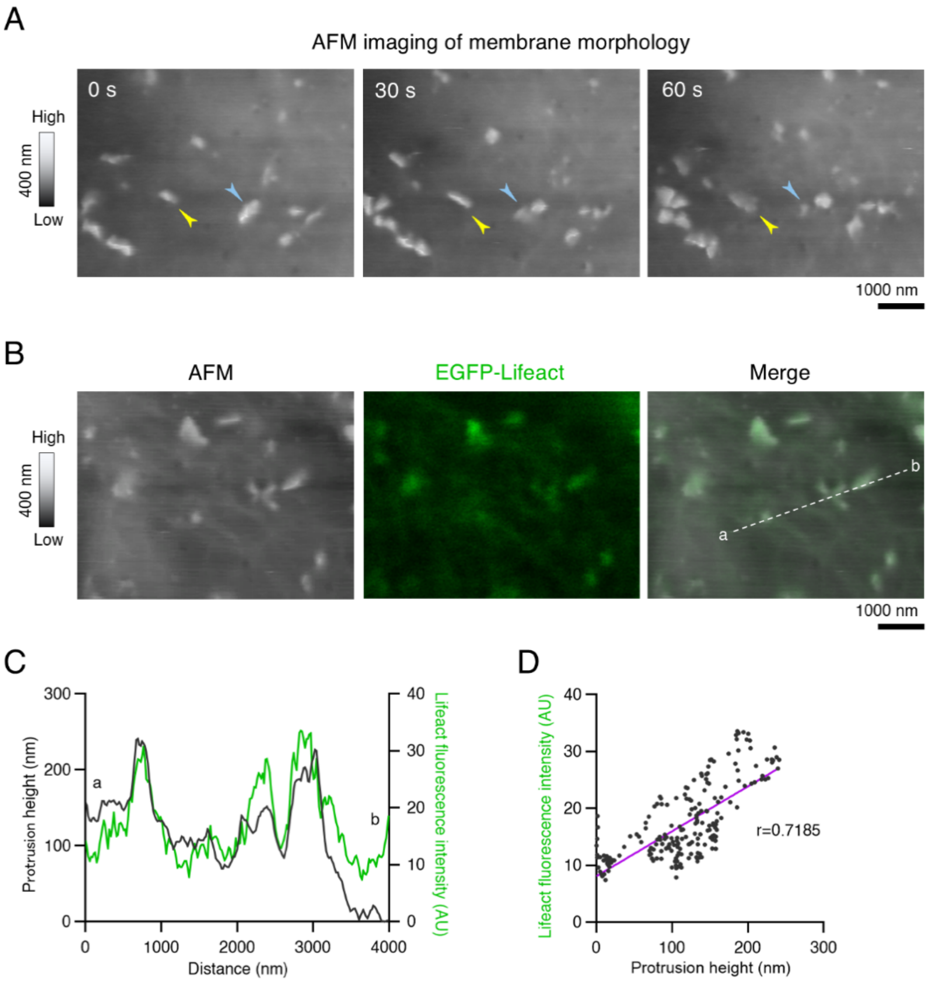


**Fig. S1.** Characterization of MDCK cell surface by ViViD-AFM (related to Fig. 1). (A) Time-lapse virus-view AFM imaging of the live MDCK cell surface. Images were acquired at 10-s intervals at 27°C, shown at 30-s intervals. Representative identical membrane protrusions are indicated by arrowheads of the same color. (B–D) Live MDCK cells expressing EGFP-Lifeact were imaged using ViViD-AFM at 27°C at 10-s intervals. (B) Representative images showing cell membrane morphology (left), Lifeact fluorescence (middle), and merged data (right). (A, B) Scale bars: 1000 nm. Height range: 400 nm. (C) Cross-sectional profile of membrane morphology (black) and Lifeact fluorescence intensity (green) along the dashed line in panel (B). The lowest height was set as 0 nm. (D) Pearson's correlation coefficient (r) between membrane height (x-axis) and Lifeact fluorescence intensity (y-axis) for the profile data in panel (C). (C, D) AU, arbitrary units.

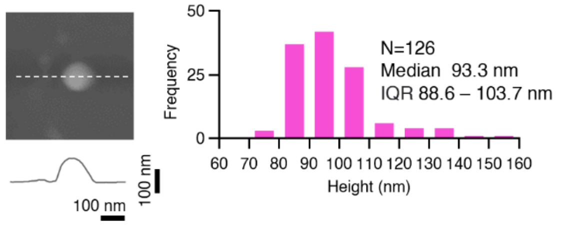


**Fig. S2.** Characterization of fluorescently labeled IAV virions on glass surface by ViViD-AFM (related to Fig. 1). IAV-AF594 virions were immobilized on fetuin-coated coverslips and were imaged at 27°C. High-magnification morphological image of a single IAV virion (top left), cross-sectional profile along the dashed line in AFM image (bottom left) and height distribution of 126 virions (right). Scale bars: 100 nm.

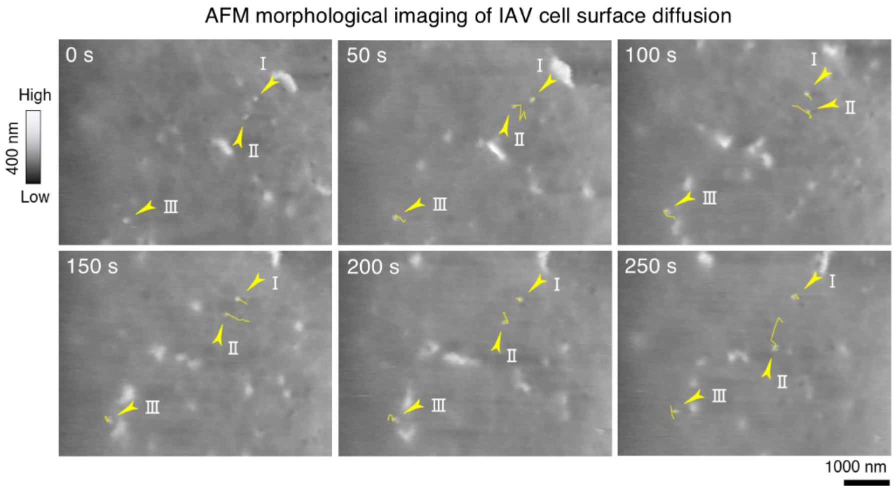


**Fig. S3.** Time-lapse morphological images of IAV cell surface diffusion (related to Fig. 1). Morphological images of cell surface IAV virions identified in Fig. 1E are shown at 50-s intervals. Arrowheads: cell surface IAV virions. Diffusion trajectories of three IAV virions during a 50-s period are superimposed on the image. Scale bar: 1000 nm. Height range: 400 nm.

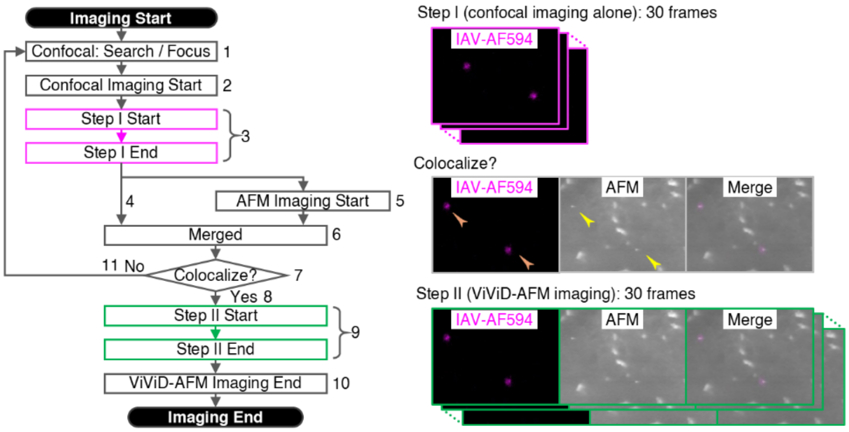


**Fig. S4.** Procedure for assessing virus-view AFM imaging effects on IAV cell surface diffusion (related to Fig. 1). Workflow for tracking identical IAV virions in Step I (confocal imaging alone) and the following Step II (ViViD-AFM imaging). (1) Initial confocal imaging focusing on IAV-AF594 fluorescent spots (magenta). (2) Time-lapse confocal imaging initiated at 27°C at 10-s intervals. (3) Collection of 30 frames (300 s) for step I. (4) Continued confocal imaging. (5) Initiation of AFM imaging concurrent with confocal imaging. (6) Overlay of confocal and AFM images. (7) Identification of virions (yellow arrowheads) co-localizing with fluorescent spots (orange arrowheads). (8) Continuation of ViViD-AFM imaging following successful co-localization. (9) Collection of 30 frames (300 s) for step II. (10) Completion of ViViD-AFM imaging. (11) If colocalization is not observed (indicating either IAV may be intracellular or the focal plane is incorrect), change observation area or readjust confocal focus and restart from Step I.

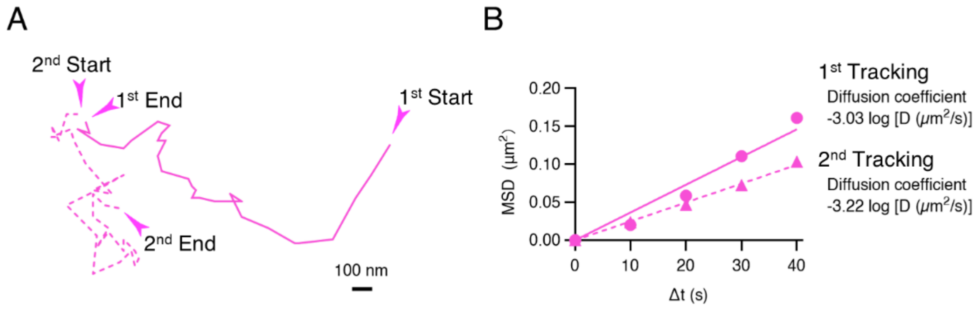


**Fig. S5.** Tracking of an identical single IAV virion in two sequential data sets acquired by confocal imaging alone (related to Fig. 1). (A) Trajectory of a single IAV/WSN-AF594 virion, captured by time-lapse confocal imaging at 27 °C at 10-s intervals. The 1st and 2nd tracking sets were collected for 300 s. (B) Quantification of IAV/WSN diffusion. Mean square displacement (MSD) plots for 1st and 2nd tracking sets from panel (A) with lines indicating two-dimensional free diffusion. The value of $\log_{10}$ [D ($\mu m^2/s$)] was calculated from MSD plots.

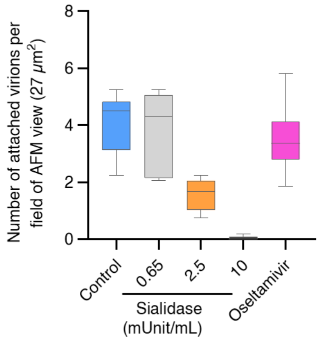


**Fig. S6.** IAV attachment in inhibitor-treated cells (related to Fig. 2). IAV/WSN-AF594 virions bound to the cell surface under control, sialidase- (0.65, 2.5, 10 mUnit/mL), and oseltamivir- (20 μM) treated conditions, counted within the AFM observation area (27 μm²) at 5 min post-inoculation at 27°C. Data are shown as median ± interquartile range.

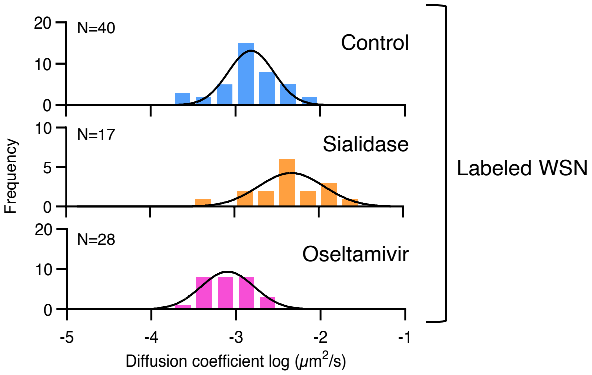


**Fig. S7.** Effect of inhibitors on IAV cell surface diffusion (related to Fig. 2). Distribution of IAV/WSN-AF594 virion diffusion coefficients on the cell surface. Top to bottom: control (N=40), sialidase-treated (N=17) and oseltamivir-treated (N=28) conditions. Data were fitted to normal distributions (same data as Fig. 2E).

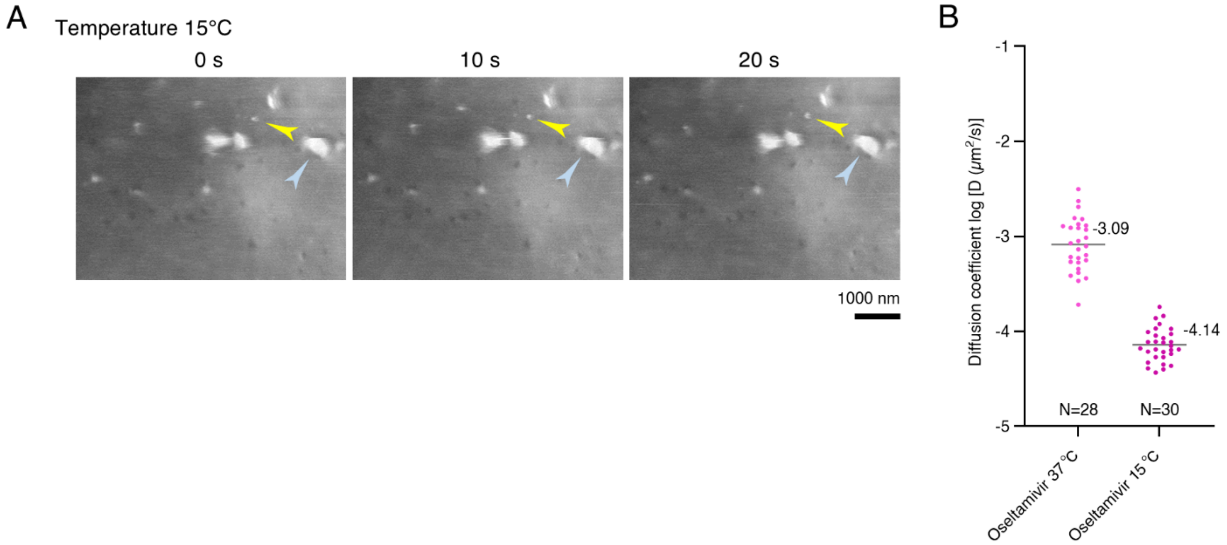


**Fig. S8.** Characterization of IAV cell surface diffusion at 15°C under Oseltamivir-treated conditions (related to Fig. 2). (A) MDCK cells were inoculated with IAV/WSN-AF594 and imaged with ViViD-AFM at 5-s intervals at 15°C. Images are shown at 10-s intervals. A representative identical membrane protrusion and an IAV virion are indicated by blue arrowheads and yellow arrowheads, respectively. Scale bar: 1000 nm. (B) Comparison of virion diffusion coefficients under oseltamivir-treated conditions at 37°C (N=28, same data as Fig. 2E) and 15°C (N=30). Bars indicate mean values.

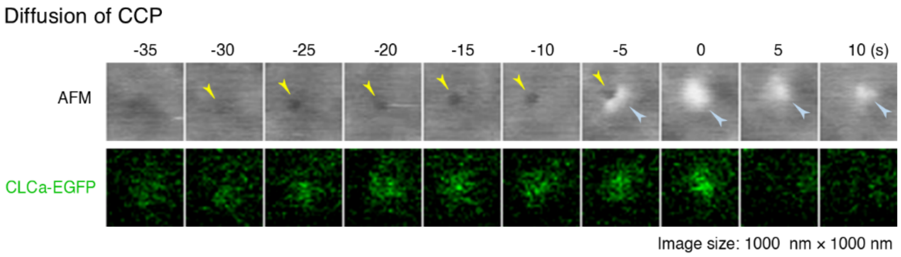


**Fig. S9.** Dynamics of virus-free clathrin-coated pits observed by ViViD-AFM (related to Fig. 3). Time-lapse imaging of clathrin-coated pit dynamics during virus-free CME from the same cell as in Fig. 3A-D. Sequential AFM (top) and CLCa-EGFP fluorescence (green, bottom) images are shown at 5-s intervals. Arrowheads indicate pit morphology (yellow) and membrane bulge morphology (blue). t = 0 s is defined as the moment when the pit becomes undetectable. Image size: 1000 × 1000 $nm^2$.

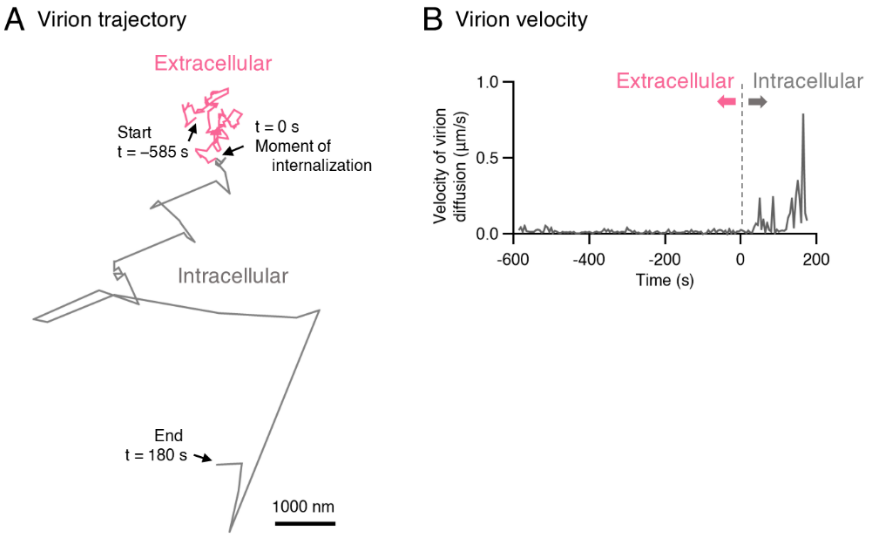


**Fig. S10.** IAV diffusion trajectory and velocity before and after internalization (related to Fig. 4A-C), captured by time-lapse ViViD-AFM imaging at 37°C at 5-s intervals. (A) Trajectory of a single IAV virion in extracellular (t = -585 s to 0 s, pink) and intracellular environments (t = 0 s to 180 s, gray). Scale bar: 1000 nm. (B) Time course of virion velocity in extracellular and intracellular environments.

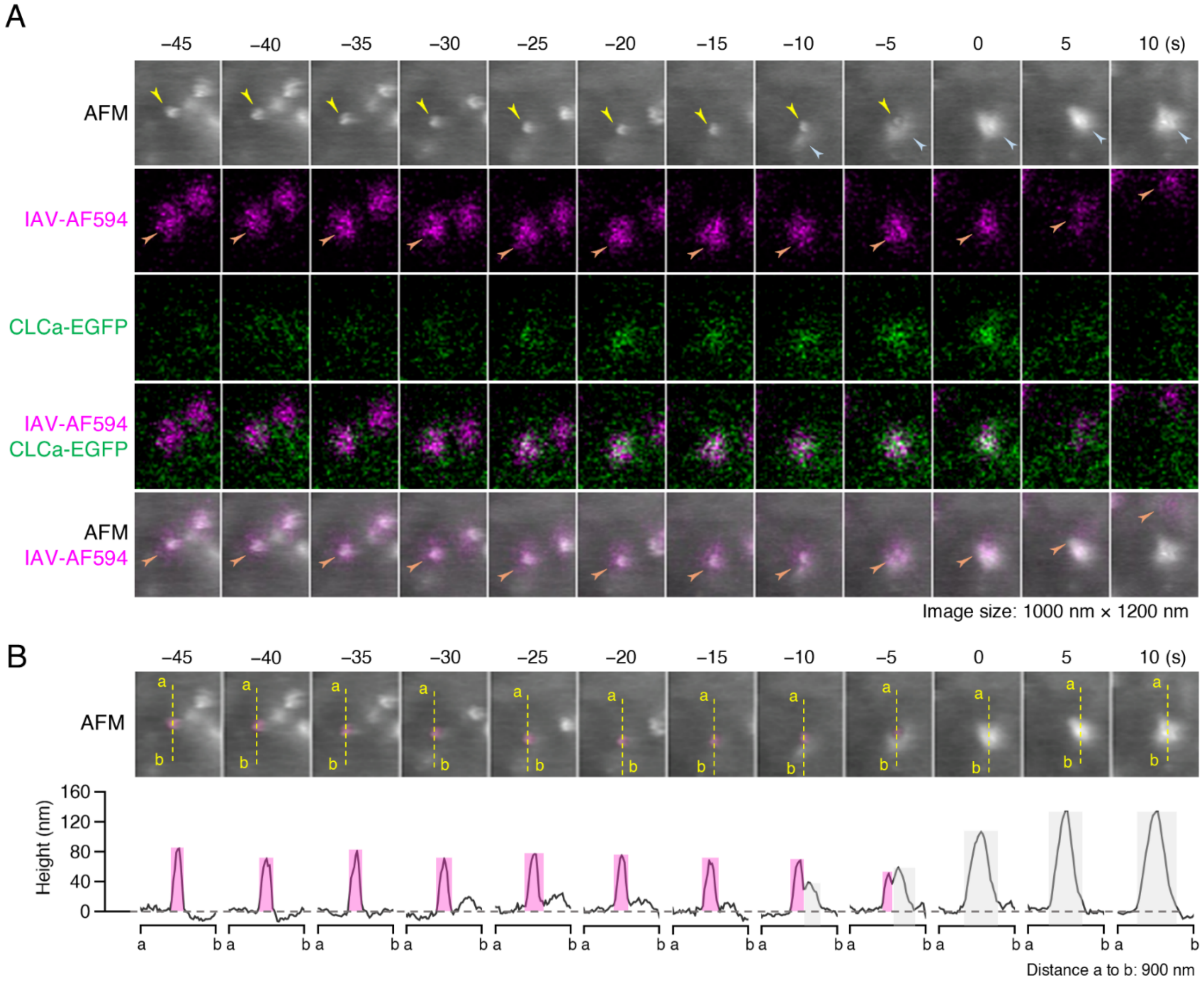


**Fig. S11.** Additional example of IAV CME captured by ViViD-AFM (related to Fig. 4). (A) IAV CME captured by time-lapse ViViD-AFM imaging at 37°C at 5-s intervals. Sequential images are shown from top to bottom: AFM, IAV-AF594 (magenta), CLCa-EGFP (green), merge of IAV-AF594 and CLCa-EGFP, and merge of AFM and IAV-AF594. Arrowheads indicate virion morphology (yellow), membrane bulges (blue) and virion fluorescence (orange). Virion morphology disappeared at t=0 s. Image size: 1000 × 1200 $nm^2$. AFM height range: 400 nm. (B) Cross-sectional profiles (bottom) along dashed lines over virion (magenta) in AFM images (top). Virions and membrane bulges in profiles are colored with magenta and gray, respectively. Virion morphology in AFM image is colored with magenta. Gray dashed line indicates membrane baseline (height = 0 nm).

Virion (–) CME

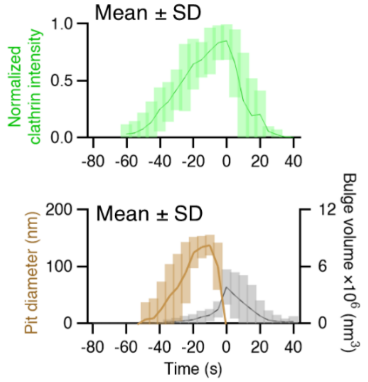


**Fig. S12.** Characterization of clathrin assembly and membrane bulges during CME without IAV (related to Fig. 4). From time-lapse ViViD-AFM data sets of 10 virion (-) CME events, including the one shown in SI Appendix, Fig. S7, fluorescence intensity of CLCa-EGFP (top, green), pit diameter (bottom, brown) and bulge size (gray, bottom) were analyzed and plotted over time. Pit morphology disappeared at t=0 s. Data are shown as mean ± standard deviation.

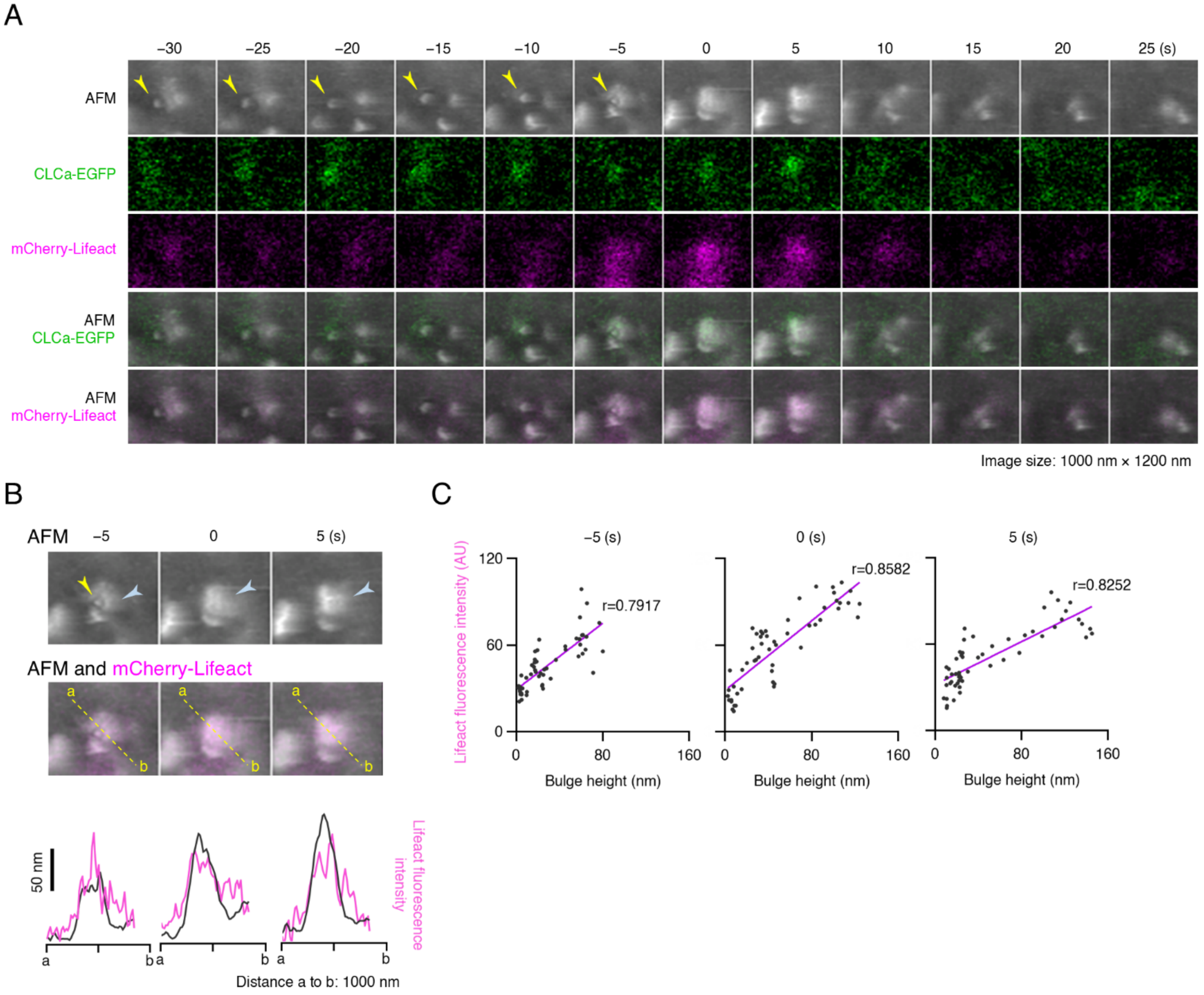


**Fig. S13.** Actin-rich membrane bulges cover virus particles in IAV CME (related to Fig. 4). MDCK cells expressing CLCa-EGFP and mCherry-Lifeact were inoculated with IAV at 37°C and was imaged by ViViD-AFM at 5-s intervals. (A) Time-lapse imaging of IAV internalization. Sequential images were shown from top to bottom: AFM, CLCa-EGFP (green), mCherry-Lifeact (magenta), merge of AFM and CLCa-EGFP, and merge of AFM and mCherry-Lifeact. t = 0 s was set as the moment when the virion morphology disappeared. Arrowheads indicate virion morphology. Image size: 1000 × 1200 $nm^2$. AFM height range: 400 nm. (B) Correlation analysis of membrane morphology and fluorescence (related to panel (A)). From top, sequential images of AFM and merge of AFM and mCherry-Lifeact (magenta) at t = -5 s, 0 s, and 5 s were shown. The lowest panel shows a plot of the cross-sectional profile of membrane morphology and Lifeact fluorescence intensity along the dashed line in the above merged image. Arrowheads indicate virion morphology (yellow), and membrane protrusions (blue). (C) Pearson's correlation coefficient (r) between membrane height (x-axis) and Lifeact fluorescence intensity (y-axis), for the profile data in panel (B). AU, arbitrary units.

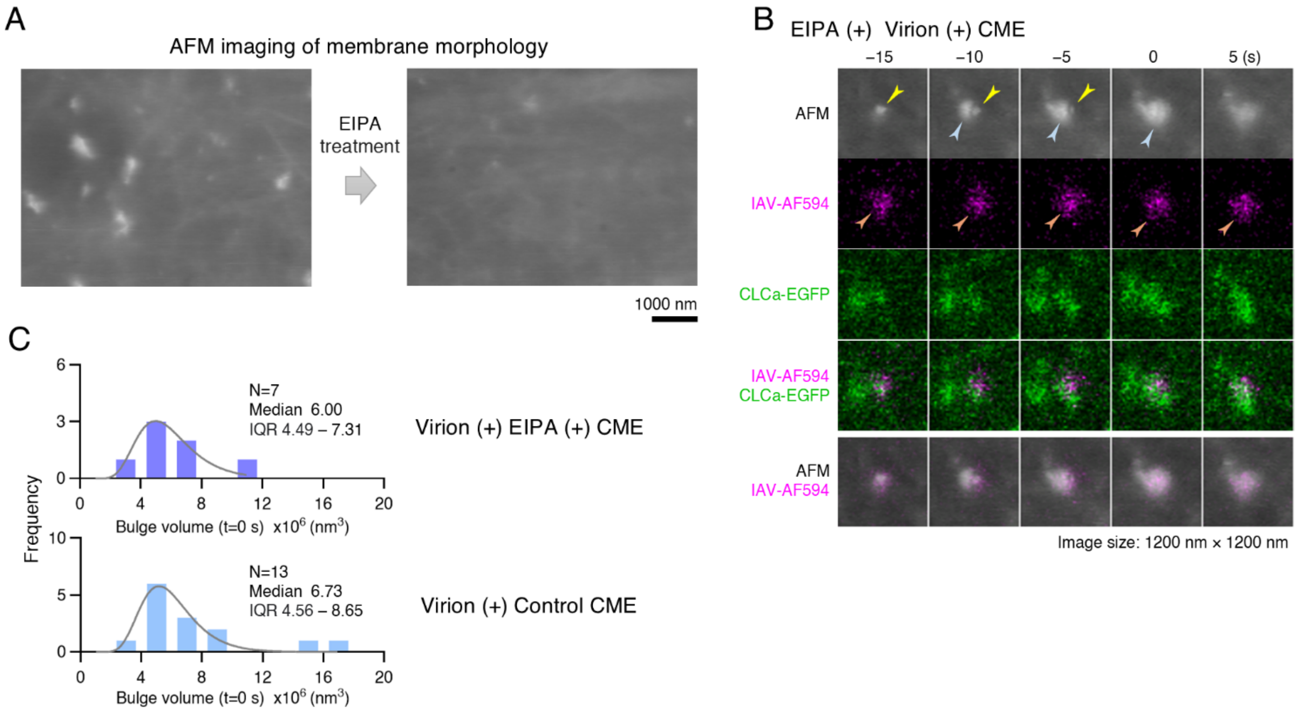


**Fig. S14.** Effects of EIPA treatment on IAV CME (related to Fig. 4). (A) Morphological images of MDCK cell surface before and after 20-min EIPA treatment at 37°C. Scale bar: 1000 nm. Height range: 400 nm. (B) Time-lapse ViViD-AFM imaging of IAV CME in MDCK cell expressing CLCa-EGFP at 37°C. From top to bottom, sequential images of AFM, IAV-AF594 (magenta), CLCa-EGFP (green), merge of IAV-AF594 and CLCa-EGFP, and merge of AFM and IAV-AF594 are shown. Arrowheads indicate virion morphology (yellow), membrane protrusions (blue) and virion fluorescence (orange). Virion morphology disappeared at t=0 s. Image size: 1200 × 1200 $nm^2$. AFM height range: 400 nm. (C) Comparison of membrane bulge volume during IAV CME between EIPA treatment (top, N=7) and without EIPA (bottom, N=13). Data were fitted using a log-normal distribution. IQR, interquartile range.

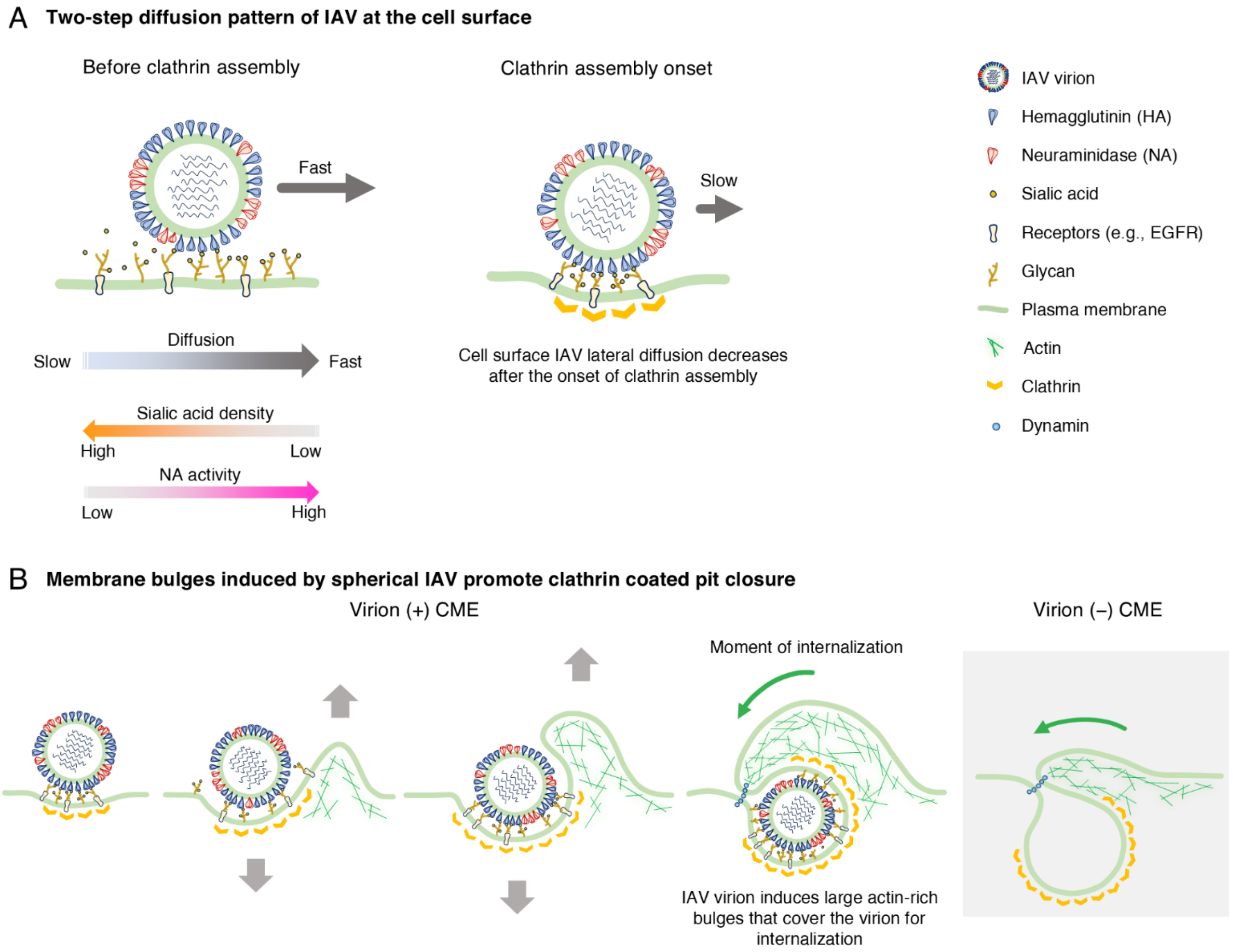


**Fig. S15.** Model of IAV diffusion and membrane dynamics during clathrin-mediated endocytosis. (A) Two-step diffusion pattern of IAV at the cell surface. Pre-clathrin assembly phase (left): IAV lateral diffusion depends on sialic acid density and neuraminidase activity - higher diffusion with lower sialic acid density, lower diffusion with reduced neuraminidase activity. Post-clathrin assembly phase (right): reduced IAV diffusion. (B) Morphological changes of cell membrane during IAV internalization. Actin-rich membrane bulges formed after clathrin assembly, simultaneously pulling the membrane up and internalizing the virion. Eventually, the virion is enveloped by membrane bulges that are larger than those observed in virus-free CME.

**Movie S1 (separate file).** Morphological imaging of the live MDCK cell surface. Time-lapse AFM imaging showing representative membrane morphological dynamics at 27°C at 10-s intervals (related to Fig. 1C and SI Appendix, Fig. S1A). The topographic AFM image displays height variations up to 400 nm, where brighter areas indicate higher regions and darker areas indicate lower regions. Image size: 6.0 × 4.5 $\mu m^2$.

**Movie S2 (separate file).** IAV lateral diffusion on the live MDCK cell surface. Time-lapse ViViD-AFM imaging showing representative IAV-AF594 virions diffusing on the cell membrane (related to Fig. 1E–F and SI Appendix, Fig. S3). Sequential images were acquired at 10-s intervals at 27°C. The movie shows the morphology of the cell surface virion (left), IAV-AF594 fluorescence (middle), and the merged image of morphology and fluorescence (right). Image size: 6.0 × 4.5 $\mu m^2$. AFM height range: 400 nm. Arrowheads indicate three virions on the cell membrane.

**Movie S3 (separate file).** Assessment of the effect of AFM imaging on IAV cell surface diffusion. Time-lapse imaging showing representative IAV cell surface diffusion in two steps (related to Fig. 1H and I), confocal imaging alone (step I) followed by ViViD-AFM imaging (step II). Sequential images were acquired at 10-s intervals at 27°C. The movie shows IAV-AF594 fluorescence (left), morphology of cell surface virion (middle), and the merge of morphology and fluorescence (right). Image size: 6.0 × 4.5 $\mu m^2$. AFM height range: 400 nm. Arrowheads indicate two virions on the cell membrane.

**Movie S4 (separate file).** IAV cell surface diffusion on live MDCK cell. Time-lapse AFM imaging showing representative IAV virion diffusion on the cell membrane from t=225 s to 575 s (related to Fig. 2A-C). Sequential images were acquired at 5-s intervals at 37°C. Arrowhead indicates the tracked virion. AFM image: 6.0 × 4.5 $\mu m^2$. AFM height range: 400 nm.

**Movie S5 (separate file).** IAV cell surface diffusion on live MDCK cell before and after clathrin assembly. Time-lapse ViViD-AFM imaging (t=0 s to 80 s) showing IAV virion diffusion on the cell membrane before and after the onset of clathrin assembly at t=50 s (related to Fig. 3A-D). Sequential images were acquired at 5-s intervals at 37°C, showing: morphology of virion and cell membrane (left), IAV-AF594 fluorescence (magenta, middle), and CLCa-EGFP fluorescence (green, right). Arrowheads indicate the virion position in AFM images, IAV-AF594 fluorescence, and the site of clathrin assembly. Image size: 6.0 × 4.5 $\mu m^2$. AFM height range: 400 nm. Clathrin assembly at the arrowhead position is observed from t=50 s.

**Movie S6 (separate file).** Moment of IAV internalization into an MDCK cell. Time-lapse AFM three-dimensional movie showing a representative IAV virion internalization event (related to Fig. 4A and B). Sequential images were acquired at 5-s intervals at 37°C. The virion was colored in magenta.

**Movie S7 (separate file).** Tracking of an IAV virion on an MDCK cell before and after CME. Time-lapse ViViD-AFM imaging showing the virion trajectory from t=-230 s to 180 s, with internalization occurring at t=0 s (related to SI Appendix, Fig. S8). Sequential images were acquired at 5-s intervals at 37°C, showing the merged fluorescence of CLCa -EGFP (green) and IAV-AF594 (magenta) (left), AFM morphology of virion and cell membrane (middle) and merged fluorescence and morphological data (right). Arrowhead indicates the virion morphology visible by AFM. In fluorescence images, the tracked virion trajectory is overlaid (orange: extracellular, gray: intracellular). Image size: 6.0 × 8.6 $\mu m^2$ (fluorescence, left) and 6.0 × 4.5 $\mu m^2$ (AFM and merged images, middle and right). AFM height range: 400 nm.

**Movie S8 (separate file).** Effect of EIPA treatment on the live MDCK cell surface morphology (related to SI Appendix, Fig. S12A). Time-lapse AFM imaging showing dynamics of the MDCK

cell surface morphology before and after 20-min EIPA treatment at 37°C. Sequential images were acquired at 5-s intervals at 37°C. Image size: 6.0 × 4.5 μm². AFM height range: 400 nm.

**Movie S9 (separate file).** IAV CME in an EIPA-treated MDCK cell (related to SI Appendix, Fig. S12B). Time-lapse AFM imaging showing representative membrane morphological changes during IAV CME, with internalization occurring at t=0 s. Sequential images were acquired at 5-s intervals at 37°C. The virion was colored in magenta. Scale bar: 1000 nm. AFM height range: 400 nm.
